# Multimodal Deep Learning for Humphrey 24-2 Visual Field Prediction: Evaluating the Contribution of Fellow-Eye Information in Glaucoma and Ocular Hypertension

**DOI:** 10.3390/jcm15187275

**Published:** 2026-09-19

**Authors:** Murat Firat, Ilknur Tuncer Firat, Haci Erbali, Emrah Ozturk, Taner Tuncer

**Affiliations:** 1 Faculty of Medicine, Malatya Turgut Özal University, 44210 Malatya, Türkiye; murat.firat@ozal.edu.tr; 2 Faculty of Medicine, Inonu University, 44280 Malatya, Türkiye; haci.erbali@inonu.edu.tr (H.E.); emrah.ozturk@inonu.edu.tr (E.O.); 3 Department of Computer Engineering, Firat University, 23119 Elazig, Türkiye; ttuncer@firat.edu.tr

**Keywords:** glaucoma, deep learning, optical coherence tomography, visual field prediction, fellow-eye interaction, bilateral modeling, structure–function relationship

## Abstract

**Background/Objectives**: To develop a multimodal deep learning framework for Humphrey 24-2 visual field prediction using multimodal OCT-derived structural information and to investigate whether incorporating fellow-eye information improves pointwise threshold sensitivity estimation in glaucoma and ocular hypertension. **Methods**: This retrospective study included 591 patients, 892 eyes, and 1099 eye-visit samples. Four OCT-derived image modalities were combined with demographic, optic disc, RNFL, and image-quality variables. Three architectures were evaluated using a patient-level 60/20/20 split: A0, a single-eye baseline; A1, bilateral Siamese feature fusion; and A2, fellow-eye-guided α-gated residual refinement applied exclusively to the 52-point threshold sensitivity output (TS52). **Results**: The baseline A0 model achieved *MAE*s of 2.97 dB (MD), 1.62 dB (PSD), and 3.26 dB (mean TS), with corresponding *RMSE*s of 4.40, 2.30, and 4.74 dB, respectively. Overall predictive performance remained comparable across architectures. Among the bilateral models, A1 achieved the lowest *RMSE* for mean threshold sensitivity (4.66 dB), whereas A2 achieved the lowest *MAE* (3.13 dB). In the paired-eye test subset (102 eyes from 51 patients), enabling fellow-eye input did not significantly improve TS52 prediction in A1 but significantly reduced TS52 RMSE (Δ = 0.08 dB, *p* = 0.0004) and MAE (Δ = 0.07 dB, *p* < 0.0001) in A2. Spatial analyses further demonstrated that A1 mainly induced a global calibration-like shift, whereas A2 selectively refined central and paracentral threshold sensitivity predictions. **Conclusions**: Fellow-eye information contributed to pointwise visual field prediction in a model-dependent manner. Within the paired-eye test subset, A2 achieved modest but statistically significant reductions in prediction error, supporting controlled residual refinement as a promising approach to incorporating fellow-eye information.

## 1. Introduction

Glaucoma comprises a group of chronic optic neuropathies characterized by progressive loss of retinal ganglion cells and their axons, structural changes in the optic nerve head, and irreversible visual field loss [1,2]. Because the disease may remain asymptomatic until substantial damage has occurred, diagnosis is often delayed. Glaucoma is therefore a leading cause of irreversible blindness worldwide, and its increasing global burden emphasizes the need for earlier detection and reliable monitoring strategies [1,2].

Structural and functional assessments are both essential in glaucoma management [2,3,4]. Standard automated perimetry, particularly the Humphrey Field Analyzer (HFA) 24-2 test, remains the clinical standard for quantifying functional loss. However, visual field testing requires subjective responses and is affected by variability and limited repeatability [2,3,4]. Optical coherence tomography (OCT), by contrast, provides quantitative structural information on the peripapillary retinal nerve fiber layer (RNFL), optic nerve head, and macular inner retinal layers. Nevertheless, the structure–function relationship in glaucoma is complex and nonlinear and is influenced by interindividual anatomical variability, disease severity, and measurement floor effects [3,4]. More accurate modeling of this relationship remains an important research objective.

Several studies have investigated the prediction of functional loss from OCT-derived structural data [5,6,7,8]. Guo et al. demonstrated that pointwise HFA 24-2 threshold sensitivities could be estimated using a nine-region OCT-based analysis [5]. Christopher et al. subsequently used deep learning to predict glaucomatous visual field damage from OCT optic nerve head en face images and RNFL thickness maps [6]. Park et al. predicted 24-2 visual fields using combined macular ganglion cell–inner plexiform layer and peripapillary RNFL measurements, whereas Lazaridis et al. improved pointwise prediction through an ensemble of deep representation learners [7,8].

These approaches have also been extended beyond cross-sectional prediction. Models based on baseline and longitudinal OCT measurements have been developed to predict subsequent visual field progression [9], while recent reviews have highlighted the expanding role of artificial intelligence in visual field prediction, pattern analysis, and progression assessment in glaucoma [10,11,12]. However, data heterogeneity, limited external and prospective validation, inadequate interpretability, and uncertain clinical generalizability remain important limitations [10,11,12]. Moreover, most existing models process each eye independently, leaving the potential value of intereye structural and topographic relationships largely unexplored.

Intereye symmetry has diagnostic value in several paired ocular structures. For example, Scheimpflug tomography demonstrates substantial intereye symmetry in healthy corneas, whereas increased asymmetry may aid the detection of keratoconus [13]. Similarly, healthy eyes exhibit a high degree of intereye symmetry in peripapillary RNFL thickness, while asymmetry beyond normal limits may indicate early glaucomatous damage [14,15]. Intereye and intraeye RNFL and macular thickness asymmetry parameters have consequently shown diagnostic value in early primary open-angle glaucoma [15]. Although glaucoma frequently progresses asymmetrically, these findings suggest that the two eyes should not necessarily be modeled as entirely independent observations. Paired visual fields in bilateral glaucoma may also exhibit complementary patterns that are not attributable to random asymmetry alone [16]. Fellow-eye information may therefore provide an integrative signal for interpreting the structure–function relationship in the target eye.

This study investigated the value of fellow-eye information for predicting HFA 24-2 visual fields from multimodal OCT-derived imaging and tabular structural data. We hypothesized that RNFL distributions and their corresponding sensitivity patterns share a partially common topographic organization between the two eyes and that fellow-eye information could improve single-eye prediction. We therefore compared a single-eye baseline model with two bilateral approaches—direct Siamese fusion and fellow-eye-guided residual refinement—and evaluated both overall predictive performance and the spatial distribution of fellow-eye effects. This bilateral framework was designed to determine whether, where, and how intereye interaction contributes to pointwise visual field prediction.

## 2. Materials and Methods

### 2.1. Study Design, Participants, and Data Acquisition

This retrospective observational study included patients with glaucoma or ocular hypertension who were followed at the Glaucoma Unit of the Department of Ophthalmology, İnönü University Turgut Özal Medical Center, between January 2024 and January 2026. The dataset comprised matched Humphrey Field Analyzer (HFA) 24-2 visual field examinations and peripapillary optical coherence tomography (OCT) optic disc scans from the same eye, together with color optic disc photographs and red-free fundus images acquired using the same device (DRI OCT Triton; Topcon Corporation, Tokyo, Japan). The study was conducted in accordance with the Declaration of Helsinki and was approved by the İnönü University Health Sciences Scientific Research Ethics Committee (approval no. 2026/10381; 30 June 2026). The requirement for informed consent was waived because of the retrospective design of the study and the use of de-identified clinical data.

Glaucoma was diagnosed based on structural findings consistent with glaucomatous optic neuropathy on fundus examination and/or OCT. These findings included neuroretinal rim thinning or notching, an increased cup-to-disc ratio, and focal or diffuse retinal nerve fiber layer (RNFL) loss. Functional confirmation was based on a reproducible visual field defect on reliable HFA 24-2 examinations. Patients aged 18 years or older with any glaucoma subtype and sufficient cooperation to complete visual field and OCT examinations were eligible. Ocular hypertension was defined as an intraocular pressure greater than 21 mmHg on at least two visits, open anterior chamber angles, no glaucomatous optic disc or RNFL damage, and a normal HFA 24-2 visual field.

Visual field examinations were performed using the Swedish Interactive Threshold Algorithm Standard (SITA Standard) strategy. Tests with fixation losses greater than 20%, false-positive errors greater than 15%, or false-negative errors greater than 15% were considered unreliable and excluded. Thus, values exactly equal to the specified thresholds were accepted.

Eligible eyes were required to have a usable HFA 24-2 examination, a peripapillary OCT optic disc scan, and the associated structural images. Visual field and OCT examinations were not required to have been performed on the same day; however, the maximum permitted interval between the OCT and visual field examinations of the same eye was four months. For participants with bilateral data, the visual field examinations of both eyes were required to have been performed on the same day, and the OCT examinations of both eyes were likewise required to have been acquired on the same day.

Exclusion criteria were unreliable visual field examinations; OCT scans with severe imaging artifacts or clinically meaningful segmentation errors; structural images in which the optic disc could not be adequately assessed; nonglaucomatous optic neuropathies; macular or retinal disorders; substantial media opacity; and additional ocular or neurological conditions that could interfere with the structure–function relationship.

No fixed lower threshold was applied to the OCT image quality score. Instead, all OCT and fundus images were individually reviewed during dataset curation, and only recordings considered inadequate for analysis were excluded. The OCT image quality score was retained as a continuous model input rather than being used solely as an exclusion criterion, thereby allowing the model to incorporate image quality as an additional signal.

For each eligible eye-visit sample, four structural image modalities were extracted: an RNFL thickness map, a circular peripapillary RNFL thickness profile, a color optic disc photograph, and a red-free fundus image. Tabular inputs included age, sex, OCT image quality score, optic disc parameters—including linear and vertical cup-to-disc ratios, rim area, disc area, and cup volume—quadrant RNFL thickness measurements, and 12 clock-hour RNFL thickness values.

The functional targets were mean deviation (*MD*), pattern standard deviation (*PSD*), and pointwise threshold sensitivity values obtained from the HFA 24-2 examination. For pointwise prediction, the two locations corresponding to the physiological blind spot were excluded from the original 54 test locations, resulting in a 52-point threshold sensitivity vector, hereafter referred to as TS52.

**Figure 1:**
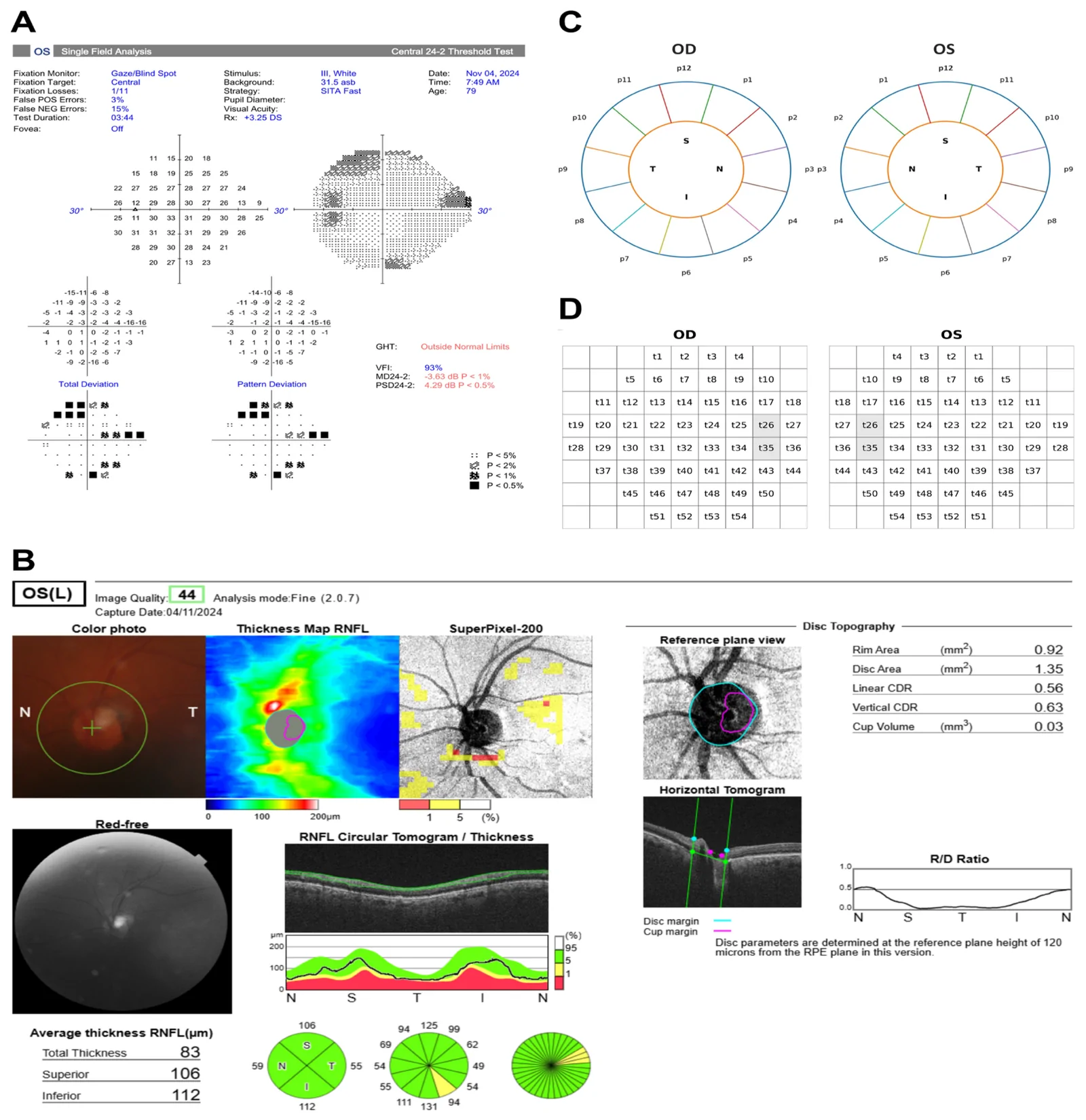
Dataset structure and right-eye canonical standardization. (**A**) Representative Humphrey Field Analyzer 24-2 visual field report. (**B**) Representative optic disc report acquired using the DRI OCT Triton device. The four image modalities used as model inputs were the color optic disc photograph, retinal nerve fiber layer (RNFL) thickness map, red-free fundus image, and circular peripapillary RNFL thickness profile. Additional panels from the original device report are shown for anatomical context but were not used as separate model inputs. (**C**) Anatomically corresponding arrangement of the 12 clock-hour peripapillary RNFL sectors in the right eye (OD) and left eye (OS). (**D**) Coding of the HFA 24-2 test locations in a right-eye canonical orientation. Left-eye locations were mirror-remapped to provide anatomical correspondence, and the two locations representing the physiological blind spot (t26 and t35) were excluded from model training and analysis. This standardization allowed right- and left-eye data to be represented within a common anatomical reference framework.

To enable anatomically comparable processing of right- and left-eye data, all measurements were transformed into a right-eye canonical orientation. Left-eye visual field locations and RNFL clock-hour measurements were mirror-remapped to their anatomically corresponding right-eye positions. Left-eye images were also horizontally flipped during data loading to establish a common spatial orientation. Each row of the primary dataset represented a single eye-visit sample identified by patient ID, eye laterality, and visit number. The dataset structure, representative HFA and OCT outputs, and the right-eye canonical standardization procedure are illustrated in Figure 1.

### 2.2. Data Preprocessing and Dataset Split

Data were indexed by patient identifier, eye laterality, and visit number. Four structural image modalities were used for each eye: the retinal nerve fiber layer (RNFL) thickness map (TM), color optic disc photograph (CP), red-free fundus image (RF), and circular peripapillary RNFL thickness profile (CT). Tabular inputs included age, sex, OCT image quality score, optic disc parameters, temporal, nasal, superior, and inferior quadrant RNFL thicknesses, and 12 clock-hour RNFL thickness measurements.

The functional targets were mean deviation (*MD*), pattern standard deviation (*PSD*), and pointwise threshold sensitivity values obtained from Humphrey 24-2 visual field examinations. To ensure anatomical comparability between right and left eyes, RNFL clock-hour measurements and visual field locations were transformed into a right-eye canonical orientation. Left-eye images were horizontally flipped during data loading to establish a common spatial orientation. The two locations corresponding to the physiological blind spot, t26 and t35, were excluded from the original 54 test locations, resulting in a 52-point threshold sensitivity vector, hereafter referred to as TS52. Visual field measurements from either eye were used only as supervised targets and evaluation references, not as predictor variables. Fellow-eye inputs were restricted to structural images and the tabular variables described above.

Before model training, patients were assigned to fixed training, validation, and test subsets in a 60%/20%/20% ratio. Both eyes and all eligible visits from each patient were retained within the same subset, with no patient overlap across partitions. The identical patient-level partition was used for A0, A1, and A2. The complete dataset comprised 591 patients, 892 unique eyes, and 1099 eye-visit samples. The training, validation, and test subsets included 355 (60.1%), 118 (20.0%), and 118 (20.0%) patients, respectively. These subsets contained 551, 172, and 169 unique eyes and 704, 226, and 169 eye-visit samples, respectively. Test patients were sampled exclusively from patients with one eligible visit; the remaining single-visit patients and all patients with multiple eligible visits were allocated to training or validation.

Fellow-eye pairing required the same patient identifier and visit number. The training, validation, and test subsets contained 232, 66, and 51 paired-eye visits from 188, 52, and 51 patients, respectively, corresponding to 464, 132, and 102 eye-visit observations. The corresponding numbers of visits with only one available eye were 240, 94, and 67.

### 2.3. Deep Learning Framework

**Figure 2:**
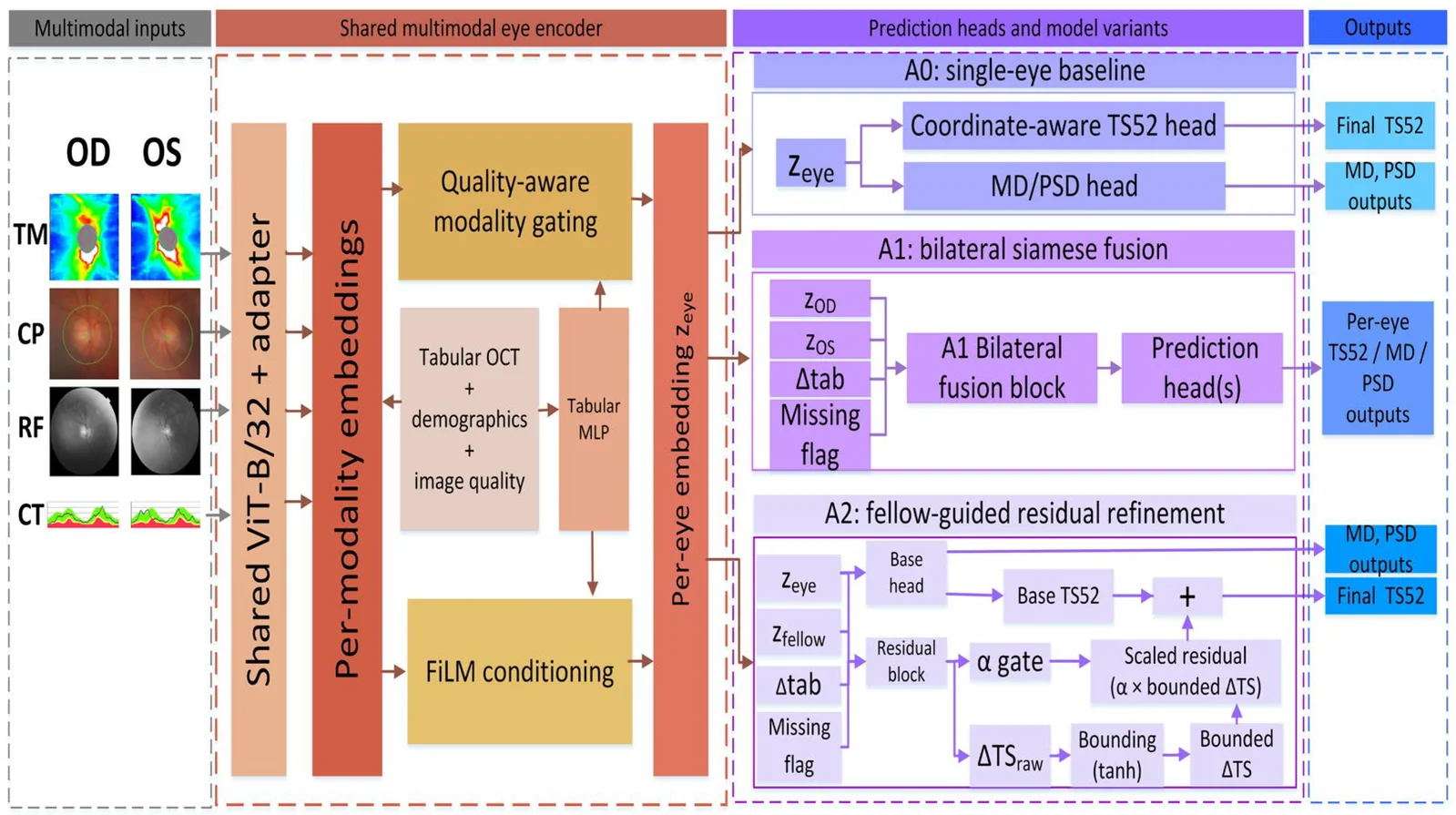
Architecture of the multimodal framework for Humphrey 24-2 visual field prediction. Thickness map (TM), color optic disc photograph (CP), red-free fundus image (RF), and circular peripapillary retinal nerve fiber layer thickness profile (CT) images from the right eye (OD) and left eye (OS) were processed using a shared multimodal eye encoder based on a Vision Transformer B/32 (ViT-B/32) backbone. Tabular OCT measurements, demographic variables, and image quality information were integrated through quality-aware modality gating and feature-wise linear modulation (FiLM), resulting in an eye-level latent representation, *z_e_*. A0 denotes the single-eye baseline model, A1 the bilateral Siamese fusion model, and A2 the fellow-eye-guided residual refinement model. In A2, an α-gated and bounded residual correction was added to the base TS52 prediction, whereas MD and PSD were predicted directly from the base branch.

The proposed framework comprised a shared multimodal encoder that learned an eye-level representation from multiple OCT-derived image modalities and associated tabular data, together with three model variants built on this representation (Figure 2). A0 served as the single-eye baseline. Both A1 and A2 incorporated fellow-eye information, using distinct mechanisms. A1 used direct bilateral Siamese feature fusion, whereas A2 applied an α-gated, fellow-eye-guided residual correction exclusively to the target-eye TS52 output. When fellow-eye data were unavailable, the A2 gate was forced to zero, leaving its base prediction unchanged. This hierarchical design allowed the contribution of fellow-eye information to be evaluated through both direct feature fusion and controlled residual refinement.

#### 2.3.1. Shared Multimodal Eye Encoder

All three model variants used the same multimodal eye encoder. A single ImageNet-pretrained ViT-B/32 backbone was shared across the four image modalities and both eyes. Each modality-specific feature representation was subsequently processed by an adapter comprising layer normalization, a linear projection, a Gaussian error linear unit activation, and dropout.

The standardized tabular variables were processed using a separate multilayer perceptron (MLP) to generate a 256-dimensional tabular embedding. This embedding was used in two complementary ways. First, it generated sample-specific modality weights through a quality-aware gating mechanism. A separate learnable modality token was combined with the tabular embedding for each image modality, and the resulting gating scores were normalized using a softmax function. Second, the tabular embedding was used to generate the scaling and shifting parameters of a FiLM conditioning block.

The OCT image quality score was therefore retained as a continuous tabular input rather than being used solely as an exclusion threshold. The eye-level latent representation was expressed as

(1) $$z_{e}=E\left(x_{e,TM},x_{e,CP},x_{e,RF},x_{e,CT},u_{e}\right)$$

where x_e,m_ denotes image modality mmm from eye e, and u_e_ denotes the standardized tabular input vector. The corresponding tabular embedding generated by the MLP is denoted by v_e_.

#### 2.3.2. A0: Single-Eye Baseline

A0 processed each target eye independently using only that eye’s inputs, regardless of whether fellow-eye data were available. Its training and evaluation included eyes from patients contributing either unilateral or bilateral data; therefore, “single-eye” describes the prediction architecture rather than a restriction to patients with unilateral data. The eye-level latent representation was passed to a coordinate-aware pointwise prediction head and a separate global prediction head:

(2) $${\hat{t}}_{k}=h_{TS}\left(\left[z_{e}∥p_{k}\right]\right),k=1,\ldots ,52,$$

(3) $$\left[{\hat{MD}}_{e},{\hat{PSD}}_{e}\right]=h_{glob}\left(z_{e}\right)$$

where p_k_ denotes the positional embedding of visual field location k, and || denotes concatenation. A0 served as the reference model for assessing whether fellow-eye information improved prediction.

#### 2.3.3. A1: Bilateral Siamese Fusion

A1 used the shared multimodal encoder in a Siamese configuration. Right- and left-eye data from the same patient-visit were processed using identical encoder weights:

(4) $$z_{OD}=E\left(x_{OD},u_{OD}\right),\;\;\;\;z_{OS}=E\left(x_{OS},u_{OS}\right)$$

For each target eye, its latent representation was combined with the fellow-eye representation, the intereye tabular difference representation, and a fellow-eye missingness indicator:

(5) $${\tilde{z}}_{e}=F\left(\left[z_{e}∥z_{f(e)}∥{\Delta v}_{e}∥m_{e}\right]\right),\;e\in \left\{OD,OS\right\},$$

where f(e) denotes the fellow eye, ∆v_e_ denotes the difference between the target-eye and fellow-eye tabular representations, and m_e_ indicates whether fellow-eye data were missing.

The fused representation was passed to the TS52 and global prediction heads:

(6) $${\hat{t}}_{e,k}=h_{TS}\left(\left[{\tilde{z}}_{e}∥p_{k}\right]\right),\;k=1,\ldots ,52,$$

(7) $$\left[{\hat{MD}}_{e},{\hat{PSD}}_{e}\right]=h_{glob}\left({\tilde{z}}_{e}\right)$$

Thus, A1 incorporated fellow-eye information directly into the fused representation used to predict TS52, MD, and PSD.

#### 2.3.4. A2: Fellow-Eye-Guided Residual Refinement

For each target eye, the fellow eye was defined as the contralateral eye of the same patient at the same visit, regardless of relative disease severity or intereye similarity. Both eyes were evaluated as targets in turn, and markedly asymmetric pairs were retained. Fellow-eye visual field measurements were not used as prediction inputs.

A2 first predicted the target eye’s visual field using that eye’s information alone. Fellow-eye information was then used to generate a correction to the 52 pointwise sensitivity estimates. A learned gating coefficient acted as a scaling factor, controlling how much of this correction was applied, while a separate bound limited its magnitude. When fellow-eye data were unavailable, the gate was set to zero, leaving the base prediction unchanged. MD and PSD were unaffected by this correction. Formally, the base branch generated pointwise threshold sensitivity estimates and global visual field indices:

(8) $$t_{e}^{base}={\left[h_{TS}\left(\left[z_{e}∥p_{k}\right]\right)\right]}_{k=1}^{52}$$

MD and PSD were therefore predicted exclusively from the base branch and were not modified by the residual refinement component.

An example-specific gating coefficient determined the extent to which fellow-eye information contributed to the residual correction:

(9) $$a_{e}=\sigma (h_{a}\left(\left(\left[\Delta v_{e}∥c_{e}∥m_{e}\right]\right)\right)\cdot \left(1-m_{e}\right)$$

where c_e_ denotes the cosine similarity between the target-eye and fellow-eye representations, and a_e_ ∈ [0, 1]. Multiplication by (1 − m_e_) forced the gate to zero when fellow-eye information was unavailable.

The unbounded TS52 residual vector was generated as

(10) $$\Delta t_{e}^{raw}=h_{\Delta }\left(\left[z_{e}∥z_{f(e)}∥\Delta v_{e}∥m_{e}\right]\right)$$

To limit excessive corrections, the residual was bounded to the interval [−10, 10] dB using a hyperbolic tangent transformation:

(11) $${\Delta t}_{e}=10tanh\left(\frac{\Delta t_{e}^{raw}}{10}\right)$$

and the final TS52 prediction was calculated as

(12) $${\hat{t}}_{e}=t_{e}^{base}+a_{e}\Delta t_{e}$$

Unlike A1, A2 did not incorporate fellow-eye information directly into the representation used for all outputs. Instead, it retained the single-eye base prediction and applied fellow-eye information as a controlled and bounded pointwise correction signal.

The gate combined differences between learned tabular embeddings, cosine similarity between multimodal eye representations, and fellow-eye availability to regulate the correction magnitude. The tabular embeddings incorporated RNFL measurements, optic disc parameters, demographic variables, and image quality; their intereye difference was not restricted to raw RNFL thickness differences. This mechanism allowed sample-specific correction without imposing a predefined relationship between disease asymmetry and gate activation.

### 2.4. Training Protocol and Regularization

All model variants were trained using a fixed random seed of 42 and an input resolution of 224 × 224 pixels. Training was performed for a maximum of 40 epochs. The mini-batch size was 16 for A0 and 8 for the bilateral A1 and A2 models because paired-eye processing required additional GPU memory.

Optimization was performed using AdamW with an initial learning rate of 2 × 10^−4^ and a weight decay of 0.05. The learning rate was reduced using a cosine annealing schedule. Training was terminated when the validation loss failed to improve for 10 consecutive epochs, and the checkpoint associated with the lowest validation loss was retained for test-set evaluation. Automatic mixed precision was enabled during GPU training. The ViT-B/32 backbone was initialized using ImageNet-pretrained weights.

Tabular variables were standardized using means and standard deviations calculated exclusively from the training subset. Missing tabular values were imputed using the corresponding training-set means. Images were converted to tensors and normalized using the standard ImageNet means and standard deviations.

Data augmentation was deliberately limited to preserve the spatial correspondence established through right-eye canonicalization. Mild brightness and contrast jitter and low-intensity Gaussian blur were applied only to the CP and RF modalities during training. No geometric augmentation was used. Horizontal flipping of left-eye images was performed as a fixed anatomical standardization step rather than as data augmentation.

For A0 and A1, the supervised loss was defined as

(13) $$L_{sup}=\lambda_{TS}L_{Huber}\left(t,\hat{t}\right)+\lambda_{MD}L_{MSE}\left(MD,\hat{MD}\right)+\lambda_{PSD}L_{MSE}\left(PSD,\hat{PSD}\right)$$

where the Huber loss was implemented as SmoothL1 loss with β = 1.0, and λ_TS_ = 1.0, λ_MD_ = 0.25, λ_PSD_ = 0.25.

A1 and A2 were trained using patient-visit samples containing either one or both available eyes. The supervised loss was averaged over the observed eyes within each sample; visits with only one available eye therefore contributed through that eye alone. In A2, the fellow-eye-dependent residual correction was disabled when fellow-eye data were unavailable.

For A2, two additional regularization terms were added:

(14) $$L_{A2}=L_{sup}+\lambda_{a}R_{a}+\lambda_{\Delta }R_{\Delta },$$

with λ_α_ = 0.01, λ_Δ_ = 0.001.

For *N_p_* paired-eye patient-visits, the gate regularization term was defined as

(15) $$R_{a}=\frac{1}{2N_{p}}\sum_{i=1}^{N_{p}}\left(a_{i,OD}^{2}+a_{i,OS}^{2}\right).$$

The residual regularization term penalized the magnitude of the actual α-scaled bounded correction rather than the raw residual:

(16) $$R_{∆}=\frac{1}{104N_{p}}\sum_{i=1}^{N_{p}}\sum_{e\in \left\{OD,OS\right\}}\sum_{k=1}^{52}{\left(a_{i,e}∆t_{i,e,k}\right)}^{2}.$$

Both regularization terms were calculated only for patient-visits in which both eyes were available. Dropout with a probability of 0.1 was used in the modality adapters, fusion and residual blocks, and prediction heads. These definitions correspond to the implemented A2 training loss. Epoch-level training and validation loss curves for the three models are provided in Figure S1.

### 2.5. Evaluation and Statistical Analysis

Model performance was evaluated on the independent test subset at both global and pointwise levels. For MD, PSD, and mean threshold sensitivity, predictive performance was quantified using mean absolute error (*MAE*), root mean square error (*RMSE*), coefficient of determination (*R*^2^), Pearson correlation coefficient (*r*), and Lin’s concordance correlation coefficient (*CCC*). Signed prediction bias was defined as the mean of predicted minus measured values.

For TS52, *MAE*, *RMSE*, *R*^2^, *r*, and *CCC* were calculated separately at each of the 52 visual field locations. Pointwise results were subsequently mapped back to the spatial Humphrey 24-2 layout and visualized as heatmaps. Agreement between measured and predicted *MD* and *PSD* values was additionally examined using Bland–Altman analysis.

Confidence intervals were estimated using patient-clustered bootstrap resampling to account for the dependency between two eyes from the same patient. Patients were resampled, and all eligible eye records belonging to each selected patient were retained together within each bootstrap sample. For a performance metric g,

(17) $$\hat{\theta }=g\left(y,\hat{y}\right),{CI}_{95\%}=\left[q_{0.025}\left({\hat{\theta }}^{*}\right),q_{0.075}\left({\hat{\theta }}^{*}\right)\right],$$

where ${\hat{\theta }}^{*}$ denotes the bootstrap distribution of the metric.

Individual model performance estimates and their confidence intervals were calculated using 2000 bootstrap replicates. Pairwise comparisons among models and fellow-eye ON/OFF analyses used 5000 bootstrap replicates.

Global pairwise comparisons among A0, A1, and A2 were performed for MD, PSD, and mean threshold sensitivity using matched eye-visit predictions. The normality of paired differences was evaluated using the Shapiro–Wilk and Kolmogorov–Smirnov tests. A paired-samples *t*-test was used when the distribution of differences was compatible with normality; otherwise, the Wilcoxon signed-rank test was applied. Patient-clustered bootstrap resampling was additionally used to estimate the mean between-model difference, its 95% confidence interval, and a two-sided bootstrap *p*-value.

Overall test-set performance was evaluated using all eligible test eyes, including those without fellow-eye data. The incremental contribution of actual fellow-eye input reported in Table 1 was assessed exclusively in the subset with both eyes available at the same visit. Samples without fellow-eye data did not contribute to this estimate.

To evaluate the direct contribution of fellow-eye information, fellow-eye ON/OFF analyses were performed for A1 and A2. Each trained model was evaluated twice. In the fellow-eye ON condition, the actual fellow-eye images and tabular data were provided. In the fellow-eye OFF condition, the fellow-eye branch was disabled using dummy image and tabular inputs together with the missingness indicator.

For each eye, TS52 *RMSE*, *MAE*, and signed prediction bias were calculated. Analyses were stratified according to whether both eyes or only one eye were available at the same patient-visit. Paired *t*-tests or Wilcoxon signed-rank tests were applied according to the distribution of the paired differences, and the comparisons were supported by patient-clustered bootstrap resampling.

For consistency across all analyses, the change in error magnitude was defined as

(18) $$∆RMSE={RMSE}_{OFF}-{RMSE}_{ON}$$

(19) $$∆MAE={MAE}_{OFF}-{MAE}_{ON}$$

Accordingly, positive ∆RMSE and ∆MAE values indicated lower prediction error when fellow-eye information was enabled. All tests were two-sided, and *p* < 0.05 was considered statistically significant.

Additional exploratory analyses included quartile-based characterization of fellow-eye benefit, correlations between fellow-eye contribution scores and Δ*RMSE*, modality-weight analyses, pointwise spatial performance mapping, and case-based patch-occlusion analysis. Detailed procedures are provided in the Supplementary Methods.

An exploratory post hoc analysis evaluated A2 performance across the predefined MD severity categories using the existing test predictions. TS52 MAE and RMSE and MD and PSD MAE were calculated within each category, with 95% confidence intervals obtained from 2000 patient-clustered bootstrap resamples. The procedure is detailed in Supplementary Methods S6, and the results are presented in Supplementary Table S7.

To provide a conventional machine-learning reference, a post hoc tabular XGBoost baseline was evaluated using the same frozen patient-level partitions. Predictors comprised the 24 target-eye demographic, optic disc, RNFL, and image-quality variables used in the deep-learning framework; images and fellow-eye information were not included. Separate regressors predicted the 52 pointwise sensitivities, MD, and PSD. Hyperparameters were selected exclusively using validation MAE, and the selected models were evaluated without refitting on the unchanged test subset. Comparisons with A0 used matched test-eye predictions and 5000 paired patient-clustered bootstrap resamples to estimate 95% confidence intervals for error differences. Detailed methods are provided in Supplementary Methods S7.

### 2.6. Implementation Environment and Additional Analyses

Deep-learning analyses were conducted using Python 3.12.10 and PyTorch 2.5.1 with CUDA 12.1. The additional tabular baseline analysis used Python 3.10.11 and XGBoost 3.1.2, as detailed in Supplementary Methods S7. Model training was conducted on an NVIDIA GeForce RTX 4050 Laptop GPU with 6 GB of memory. The complete hardware and software environment is provided in Table S1.

## 3. Results

### 3.1. Cohort and Dataset Characteristics

The final dataset comprised 591 patients, 892 unique eyes, and 1099 eye-visit samples. Across all eligible visits, 301 patients (50.9%) contributed data from both eyes, and 290 (49.1%) contributed data from only one eye. Of the 301 patients contributing bilateral data, 291 had at least one visit with both eyes available, whereas 10 contributed right- and left-eye data at different visits without a same-visit pair. Cohort composition and dataset characteristics are summarized in Table 1.

### 3.2. Global Test-Set Performance

Global test-set performance is summarized in Table 2. Differences in *MAE* across models were generally limited. Both bilateral models showed lower positive mean threshold sensitivity bias than A0, whereas A1 and A2 differed in the direction of their *MD* and *PSD* bias. Complete pairwise comparisons are presented in Table S2. Bland–Altman analyses showed broadly comparable limits of agreement across models and are provided in Figure S2.

In the exploratory severity-stratified analysis, A2 prediction errors were higher in more advanced visual field loss (Supplementary Table S7). TS52 MAE was 2.63 dB in the early group, 2.79 dB in the mild group, 5.45 dB in the moderate group, and 10.13 dB in the severe group. Among the 22 severe eyes from 20 patients, TS52 RMSE was 12.47 dB and MD MAE was 6.81 dB.

The tabular XGBoost baseline yielded numerically lower errors than A0, although all paired difference confidence intervals included zero (Supplementary Table S8). TS52 MAE was 3.92 versus 3.99 dB, with an XGBoost-minus-A0 difference of −0.07 dB (95% CI, −0.29 to 0.17). Pooled TS52 RMSE was 6.16 versus 6.38 dB, with a difference of −0.23 dB (95% CI, −0.53 to 0.10). Differences in MD, PSD, and mean threshold sensitivity MAE were also uncertain.

### 3.3. Direct Effect of Fellow-Eye Input on Pointwise Prediction

The same-visit paired-eye test subset comprised 102 eyes from 51 patients and formed the basis of the fellow-eye ON/OFF comparisons reported in Table 3. In A1, enabling fellow-eye input did not significantly reduce TS52 RMSE or MAE but produced a significant shift in signed bias. In A2, fellow-eye input significantly reduced TS52 RMSE, MAE, and signed bias (Table 3).

### 3.4. Spatial Pattern and Magnitude of Fellow-Eye Contribution

**Figure 3:**
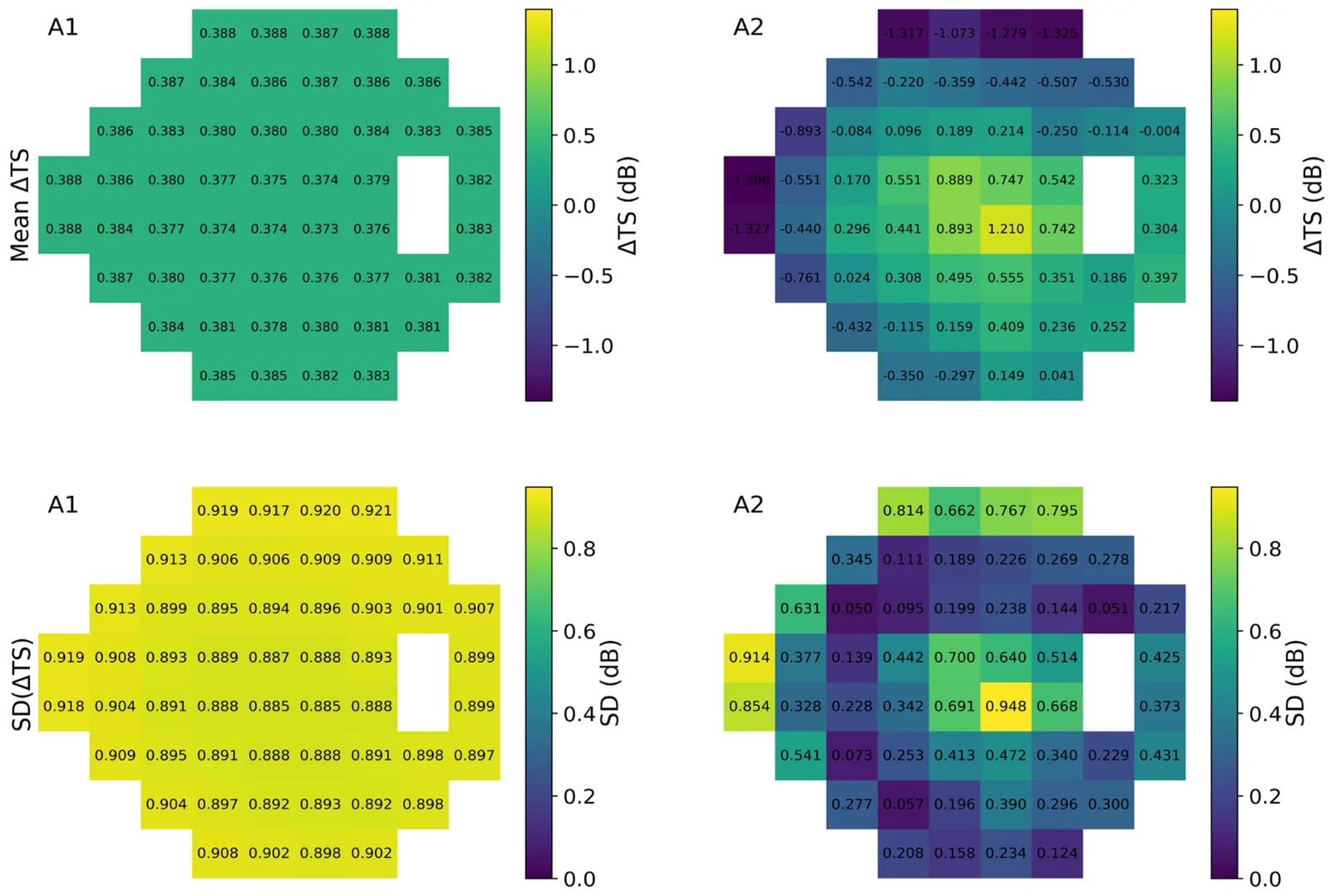
Spatial pattern of fellow-eye contribution to TS52 predictions in paired-eye test samples.

**Figure 4:**
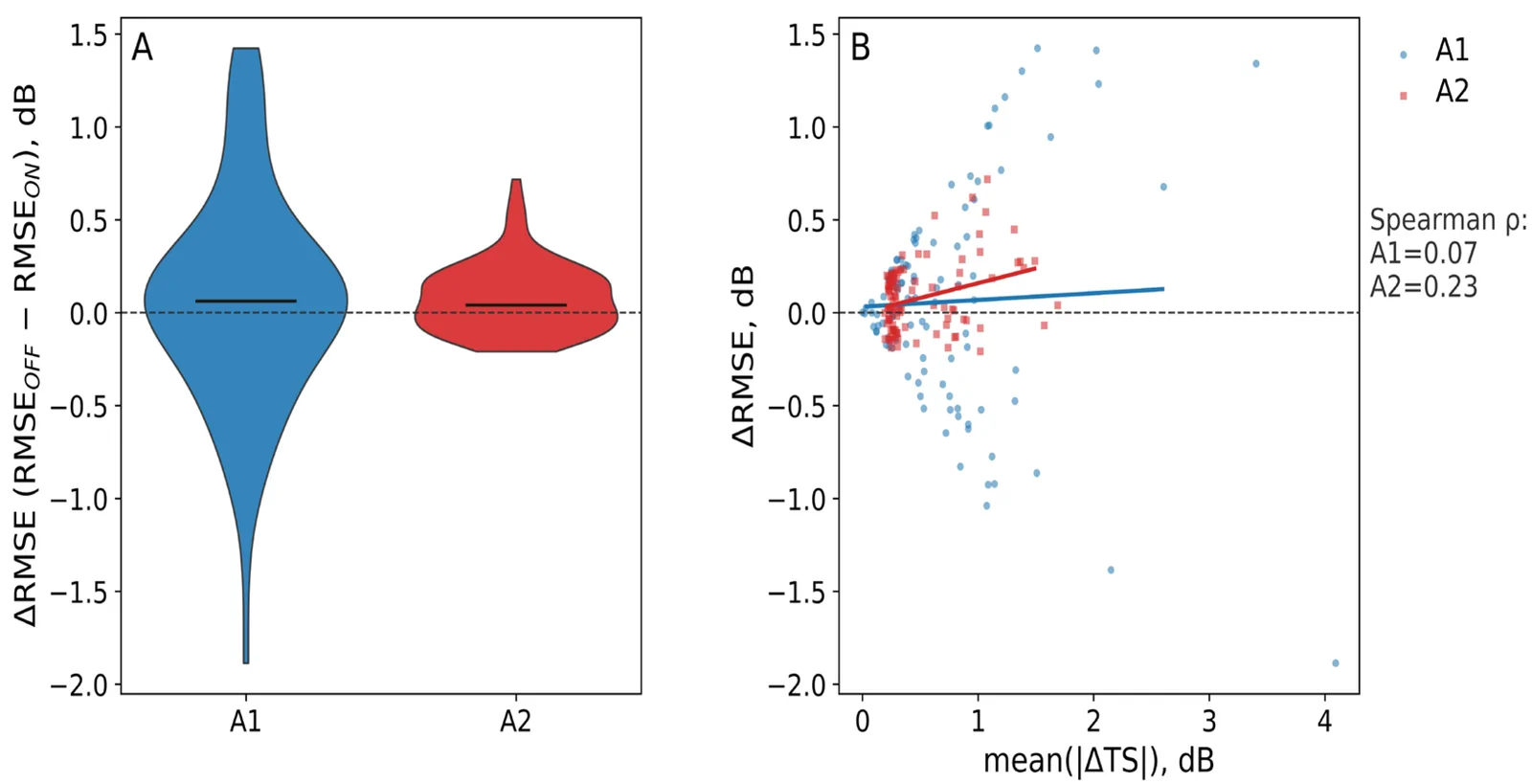
Magnitude of fellow-eye contribution and its association with improvement in prediction error. (**A**) Distribution of $∆RMSE={RMSE}_{OFF}-{RMSE}_{ON}$ for A1 and A2. Positive values indicate lower error when fellow-eye information was enabled. (**B**) Association between mean absolute Δ*TS* and Δ*RMSE*. No significant association was observed for A1 (ρ = 0.07), whereas A2 showed a weak positive association (ρ = 0.23).

In exploratory quartile analyses (Figure 3), the greatest A2 benefit was observed among eyes with worse *MD*, lower mean threshold sensitivity, and larger intereye structural and functional differences, whereas the A1 pattern was less consistent (Table S3). Mean absolute Δ*TS* was not associated with Δ*RMSE* in A1 but showed a weak positive association in A2. The A2 α-gate value itself was not significantly associated with Δ*RMSE* (Table S4 and Figure 4). In A2, Δ*RMSE* was positively associated with the TM and CT weights and negatively associated with the CP and RF weights; however, the variability of these normalized weights was very small (Table S5).

For each eye, Δ*TS* was calculated as *TS_ON_* − *TS_OFF_* and mapped to the right-eye canonical 24-2 layout. The upper row shows the mean ΔTS maps and the lower row shows the standard deviation of Δ*TS* for A1 and A2. Positive values indicate higher predicted sensitivity when fellow-eye input was enabled.

### 3.5. Additional Pointwise and Explainability Analyses

The overall central and paracentral threshold sensitivity pattern was retained across all models, although predicted values showed compression toward the mean at lower-sensitivity locations (Figure S3). Pointwise error and agreement patterns were spatially heterogeneous but broadly similar across models, with localized differences in selected test locations (Figures S4 and S5). In the two illustrative paired-eye cases, patch-occlusion maps appeared more diffuse in A1 and more compact in A2 (Figure S6).

## 4. Discussion

In this study, we compared three architectures to assess whether fellow-eye information could improve multimodal prediction of Humphrey Field Analyzer 24-2 visual field outcomes. A0 served as the single-eye reference model, A1 incorporated fellow-eye information through direct bilateral Siamese fusion, and A2 used the fellow eye only to generate an α-gated and bounded residual correction for the TS52 output. Differences in global performance were generally small. A1 achieved the lowest MAE for MD and PSD and the lowest RMSE for mean threshold sensitivity, whereas A2 achieved the lowest MAE for mean threshold sensitivity. Pairwise analyses indicated that the global effect of bilateral modeling was more apparent in prediction bias than in consistent reductions in error magnitude. In the paired-eye ON/OFF analysis, A2 produced statistically significant but small reductions in TS52 RMSE and MAE, whereas A1 did not significantly reduce either error measure. These findings suggest that the contribution of fellow-eye information was model-dependent and was more evident for pointwise prediction than for global visual field indices.

Selected studies of imaging-based 24-2 visual field prediction and their reported performance are summarized in Table S6. Guo et al. [5] used regional structure–function mapping to estimate individual 24-2 thresholds, whereas Christopher et al. [6] used optic nerve head en face images and RNFL thickness maps to predict global and sectoral visual field outcomes. Park et al. [7] predicted the 52-point 24-2 field from combined macular GCIPL and peripapillary RNFL thickness maps, and Lazaridis et al. [8] used an ensemble of deep representation learners to estimate pointwise visual function. Subsequent studies expanded the range of inputs and fusion strategies. Kihara et al. [17] introduced policy-driven multimodal fusion of OCT and infrared optic disc images, Hemelings et al. [18] used unsegmented OCT scans for pointwise estimation, and Christopher et al. [19] estimated 24-2 global indices from macular retinal layer thickness maps.

More recent approaches have examined whether additional information or alternative representations can improve structure–function modeling. Chen et al. [20] reported improved pointwise estimation using segmentation-free three-dimensional OCT volumes. Sriwatana et al. [21] incorporated artifact correction before transformer-based prediction, emphasizing the influence of OCT image quality. Mahmoudinezhad et al. [22] showed that optic nerve head OCT angiography could also be used to estimate 24-2 outcomes. Chuangsuwanich et al. [23] combined optic nerve head morphology with IOP-induced neural tissue strain and reported improved classification of visual field defect locations compared with structural information alone. Because these studies differed substantially in their outcomes and test populations, their numerical results should not be interpreted as direct head-to-head comparisons.

The additional XGBoost comparison provided a conventional tabular reference under the same patient-level partition. XGBoost produced numerically lower errors than A0, but the paired confidence intervals included zero for all evaluated differences. These findings do not establish superiority of either approach or statistical equivalence. Because the models differed in both input modalities and learning algorithms, the comparison cannot isolate the effect of architecture or quantify the added value of images.

The present study extends this literature in two main respects. First, four structural image modalities were integrated with demographic, optic disc, RNFL, and image-quality variables within a common multimodal encoder. Second, fellow-eye information was evaluated using two distinct bilateral strategies rather than being treated as an additional undifferentiated input. Direct Siamese fusion and gated residual refinement were trained and evaluated using the same patient-level split and broadly similar optimization procedures. This design reduced some sources of variation between the models and allowed the effect of the bilateral processing strategy to be examined more directly. Nevertheless, the observed differences remained small and should not be interpreted as evidence that bilateral modeling is uniformly superior to single-eye prediction.

The rationale for incorporating fellow-eye information is supported by previous observations of interocular structural and functional relationships. Mwanza et al. [14] reported substantial interocular symmetry in peripapillary RNFL thickness among healthy eyes. Sullivan-Mee et al. [15] subsequently showed that interocular and intraocular asymmetry measures could contribute to the identification of early primary open-angle glaucoma. Functional findings also indicate that the two eyes are related but not interchangeable. Sponsel et al. [16] described bilateral visual field patterns that were compatible with a nonrandom organization of residual binocular function. Teng et al. [24] demonstrated associations between better- and worse-eye visual field defects and evaluated the clinical utility of fellow-eye information. Boden et al. [25] reported partial, rather than complete, spatial correspondence between visual field defects in the two eyes, and Bak et al. [26] associated greater intereye visual-sensitivity asymmetry with faster visual field progression. Yousefi et al. [27] further characterized asymmetric hemifield patterns in open-angle and angle-closure glaucoma. At the structural level, Shoji et al. [28] proposed an automated temporal raphe-based framework for measuring macular asymmetry. Together, these studies support the use of interocular information as contextual input, while also indicating that simple mirror symmetry is unlikely to describe the full bilateral relationship.

The comparison between A1 and A2 suggests that the manner in which this contextual information is introduced may be important. Siamese networks provide a general framework for learning relationships between paired medical images and have been used for disease severity estimation, longitudinal change analysis, and glaucoma-related classification [29,30,31]. More broadly, multimodal fusion and attention-based methods provide mechanisms for combining heterogeneous inputs while regulating their relative contribution [32,33]. These studies provide methodological precedents rather than direct evidence that a particular bilateral architecture should perform better in glaucoma.

In A1, the target-eye representation was directly fused with the fellow-eye representation and intereye tabular differences before prediction. The ON/OFF analysis indicated that this approach primarily altered TS52 calibration, producing a significant bias shift without a significant reduction in *RMSE* or *MAE*. Its broader Δ*RMSE* distribution also suggested greater variability in the effect of fellow-eye input across eyes.

A2 instead preserved the single-eye base prediction and restricted fellow-eye information to an α-gated, bounded TS52 correction. In the paired-eye subset, enabling fellow-eye input reduced *RMSE* by 0.08 dB and *MAE* by 0.07 dB. The narrower Δ*RMSE* distribution and the association between the magnitude of fellow-eye-induced TS change and RMSE improvement were consistent with a more controlled use of bilateral information. The difference between pointwise and global results partly reflects the architecture: fellow-eye information directly refined TS52, whereas MD and PSD were predicted from the target-eye base branch and were not recalculated from the refined sensitivities. Improvements in TS52 therefore need not translate into improvements in these global indices. Opposing changes across individual locations may also yield little net change in mean threshold sensitivity.

The exploratory quartile and spatial analyses further distinguished the two architectures. A1 benefit was more apparent in eyes with relatively preserved function and smaller intereye differences, whereas the greatest A2 benefit occurred in eyes with worse *MD*, lower mean threshold sensitivity, and greater structural and functional asymmetry (Table S3). A1 produced a more uniform shift across the 24-2 field, while A2 generated a more heterogeneous pattern with greater changes at selected central and paracentral locations. However, α itself was not significantly associated with Δ*RMSE*, indicating that greater gate activation did not necessarily produce greater benefit (Table S4). The illustrative patch-occlusion examples, shown in Figure S6, similarly demonstrated a more diffuse importance pattern for A1 and a more compact pattern for A2. Although exploratory, these findings were consistent with the architectural distinction between direct fusion and residual refinement.

Although several A2 modality weights were significantly associated with Δ*RMSE*, their between-eye variability was very small. Among the four modalities, Δ*RMSE* showed the strongest absolute association with the RF weight (ρ = −0.345), closely followed by the TM weight (ρ = 0.342); CT was also positively associated, whereas CP showed a negative association. This pattern may suggest that greater emphasis on RNFL-derived structural representations, particularly TM and CT, was more compatible with beneficial fellow-eye refinement, while the inverse associations for CP and RF may partly reflect the competitive, sum-to-one gating mechanism rather than lower intrinsic informativeness of fundus images. The correlations should therefore be interpreted cautiously, as they may reflect subtle variation within a narrow range rather than substantial sample-specific modality reweighting. Detailed modality-weight estimates and their correlations with Δ*RMSE* are provided in Table S5. These analyses do not separate the contributions of image-derived and tabular information. Tabular features influenced both the multimodal eye representations and the intereye correction pathway, preventing attribution of the observed gain to RNFL differences or image features alone. Targeted component-ablation analyses would be required to distinguish these contributions.

Fellow-eye modeling should be considered complementary to, rather than a replacement for, primary-eye information. It may provide additional context when visual field measurements are noisy, incomplete, or inconsistent with structural findings. Beyond direct field estimation, fellow-eye information could be incorporated into quality-control systems, asymmetry-aware calibration modules, and repeat-testing algorithms to identify predictions that are unstable or discordant with bilateral structural patterns. It may also support longitudinal monitoring by providing an internal reference for detecting changes that are unexpected in relation to the fellow eye. A further potential application is prediction when target-eye structural images are of limited quality, for example because of media opacity, while fellow-eye images remain informative. However, the average reductions of 0.07 dB in MAE and 0.08 dB in RMSE do not establish a clinically actionable improvement for individual patients. These reductions in prediction error should not be interpreted as evidence of detectable functional change, and marked intereye differences may further limit the usefulness of fellow-eye information. These proposed applications, including longitudinal monitoring, were not directly evaluated in the present study.

This study has several limitations. It was retrospective, single-center, and based on a single OCT platform, without external or prospective validation. External validation across centers and OCT platforms is needed to assess robustness to differences in patient populations, image acquisition, and device-specific measurements before clinical implementation. Such evaluation should examine both overall prediction accuracy and the reproducibility of the incremental benefit from fellow-eye information. Early and mild visual field loss predominated, so aggregate performance largely reflects these eyes, while fewer advanced cases were available for learning and evaluation. The exploratory severity-stratified analysis showed substantially higher TS52 errors in severe disease (Supplementary Table S7), indicating that overall accuracy should not be generalized to advanced glaucoma. A similar severity-related increase in prediction error was reported for multimodal OCT–fundus prediction of HFA 30–2 visual fields [34]. Structural measurement floors and greater functional variability may contribute to this difficulty, although their individual effects were not isolated here. Our severe subgroup comprised only 22 eyes from 20 patients, warranting further evaluation in a larger advanced-disease cohort. The fellow-eye ON/OFF analysis was restricted to 102 eyes from 51 patients with same-visit bilateral data. This limited paired-eye sample size constrains the precision and generalizability of the estimated fellow-eye effects, particularly across disease-severity and intereye-asymmetry subgroups. These comparisons assessed the dependence of trained models on fellow-eye input rather than the effect of training separate models with and without such information. In addition, test patients were selected exclusively from those with one eligible visit, which may limit the representativeness of the test cohort for patients undergoing repeated follow-up. Spatial and case-based explainability analyses were exploratory, and the low variability in modality weights limits their interpretation. Finally, severity-aware sampling and selective augmentation were not used in order to maintain a more comparable training framework across A0, A1, and A2, although this may have limited absolute model performance.

## 5. Conclusions

This study showed that fellow-eye information can contribute to multimodal prediction of Humphrey 24-2 visual fields, with its effect depending on how bilateral information is incorporated. Direct Siamese fusion primarily produced a calibration-like shift, whereas α-gated residual refinement yielded more selective, spatially heterogeneous changes and modest but statistically significant reductions in pointwise prediction error within the paired-eye test subset. These findings support controlled residual refinement as a promising strategy for using fellow-eye information as a contextual correction signal while retaining the target eye as the basis for prediction. This approach warrants further investigation to determine the settings in which bilateral structural information provides the greatest predictive value. Independent multicenter validation across OCT platforms is a prerequisite for any clinical deployment, and prospective evaluation should be a priority for future work.

## Figures and Tables

**Table 1:** Cohort composition and dataset characteristics.

Characteristic	Value
**Dataset structure**	
Patients, n	591
Unique eyes, n	892
Eye-visit samples, n	1099
Patients contributing bilateral data, n (%)	301 (50.9)
Patients contributing unilateral data, n (%)	290 (49.1)
**Visit structure**	
Distinct patient-visits, n	750
Patient-visits with both eyes available, n (%)	349 (46.5)
Patient-visits with one eye available, n (%)	401 (53.5)
Patients with one eligible visit, n (%)	446 (75.5)
Patients with two eligible visits, n (%)	131 (22.2)
Patients with three eligible visits, n (%)	14 (2.4)
**Patient-level demographics**	
Age, years	57.86 ± 16.24
Female patients, n (%)	302 (51.1)
Male patients, n (%)	289 (48.9)
**Eye-visit laterality**	
Right-eye samples, n (%)	542 (49.3)
Left-eye samples, n (%)	557 (50.7)
**Visual field severity according to MD**	
Early, n (%)	598 (54.4)
Mild, n (%)	253 (23.0)
Moderate, n (%)	135 (12.3)
Severe, n (%)	113 (10.3)
**Visual field metrics**	
VFI, %	90.51 ± 15.99
MD, dB	−4.27 ± 5.63
PSD, dB	3.31 ± 2.81
Mean TS52, dB	25.92 ± 5.83
Mean TD52, dB	−4.34 ± 5.68
Mean PD52, dB	−2.98 ± 2.45
**Visual field reliability indices**	
Fixation losses, %	2.61 ± 4.96
False-positive errors, %	2.42 ± 3.28
False-negative errors, %	3.48 ± 4.96
**OCT measurements**	
OCT image quality score	44.70 ± 8.59
Temporal RNFL thickness, µm	66.62 ± 15.38
Nasal RNFL thickness, µm	74.51 ± 17.72
Superior RNFL thickness, µm	110.86 ± 29.96
Inferior RNFL thickness, µm	114.49 ± 33.28
Mean clock-hour RNFL thickness, µm	91.55 ± 20.69

Data are presented as n (%) or mean ± standard deviation. Age, sex, bilateral/unilateral data contribution, and the number of eligible visits per patient were summarized at the patient level (n = 591). Patient-visits with one or both eyes available were calculated using 750 distinct patient-visits. Eye laterality, visual field characteristics, and OCT measurements were summarized at the eye-visit level (n = 1099). Visual field severity was categorized according to MD as follows: early, MD > −3 dB; mild, −6 dB < MD ≤ −3 dB; moderate, −12 dB < MD ≤ −6 dB; and severe, MD ≤ −12 dB. Patient-level bilateral data contribution indicates that both eyes were represented across eligible visits and does not necessarily indicate same-visit availability. Of the 301 patients contributing bilateral data, 291 had at least one same-visit eye pair.

**Table 2:** Global test-set performance for MD, PSD, and mean threshold sensitivity across the three models.

Outcome	Model	Predicted Value, Mean ± SD	MAE [95% CI]	RMSE [95% CI]	CCC [95% CI]	r [95% CI]	Bias
MD	A0	−4.53 ± 4.30	2.97 [2.49, 3.52]	4.40 [3.54, 5.30]	0.62 [0.46, 0.75]	0.65 [0.51, 0.77]	−0.39
MD	A1	−4.43 ± 4.27	2.90 [2.39, 3.46]	4.39 [3.52, 5.27]	0.62 [0.46, 0.75]	0.65 [0.51, 0.77]	−0.28
MD	A2	−3.83 ± 3.94	2.93 [2.43, 3.50]	4.42 [3.50, 5.32]	0.59 [0.43, 0.73]	0.64 [0.48, 0.77]	0.31
PSD	A0	3.42 ± 1.98	1.62 [1.33, 1.91]	2.30 [1.84, 2.72]	0.60 [0.46, 0.73]	0.66 [0.53, 0.78]	−0.03
PSD	A1	3.20 ± 2.02	1.56 [1.24, 1.89]	2.38 [1.89, 2.82]	0.58 [0.44, 0.70]	0.64 [0.51, 0.75]	−0.25
PSD	A2	3.40 ± 1.92	1.66 [1.36, 1.96]	2.41 [1.95, 2.82]	0.56 [0.42, 0.68]	0.62 [0.48, 0.74]	−0.05
Mean TS	A0	27.26 ± 4.29	3.26 [2.71, 3.83]	4.74 [3.89, 5.59]	0.60 [0.46, 0.72]	0.65 [0.53, 0.76]	1.25
Mean TS	A1	26.67 ± 3.38	3.21 [2.68, 3.78]	4.66 [3.85, 5.48]	0.55 [0.41, 0.67]	0.65 [0.53, 0.76]	0.66
Mean TS	A2	26.74 ± 3.40	3.13 [2.59, 3.69]	4.71 [3.81, 5.55]	0.54 [0.40, 0.67]	0.64 [0.51, 0.76]	0.73

**Table 3:** Effect of fellow-eye input on TS52 error metrics in same-visit paired-eye test samples (102 eyes from 51 patients).

Model	Metric	Δ (OFF − ON), dB	95% CI	Bootstrap *p*	Paired-Test *p*
A1	TS52 RMSE (dB)	0.06	[−0.07, 0.19]	0.3612	0.2721
A1	TS52 MAE (dB)	0.06	[−0.06, 0.19]	0.3288	0.0980
A1	TS52 Bias (dB)	−0.38	[−0.52, −0.23]	<0.0001	<0.0001
A2	TS52 RMSE (dB)	0.08	[0.04, 0.12]	0.0004	0.0001
A2	TS52 MAE (dB)	0.07	[0.03, 0.11]	<0.0001	0.0002
A2	TS52 Bias (dB)	0.07	[0.05, 0.08]	<0.0001	<0.0001

## Data Availability

The data supporting the findings of this study are available from the corresponding author upon reasonable request. The data are not publicly available because they contain clinical information and are subject to institutional and ethical restrictions.

## Supplementary Materials

The following supporting information can be downloaded at: https://www.mdpi.com/article/10.3390/jcm15187275/s1, Supplementary Methods: Additional exploratory and explainability analyses; Figure S1: Epoch-level training and validation loss curves for the A0, A1, and A2 models; Table S1: Hardware and software environment used for model training and evaluation; Table S2: Global pairwise differences in MAE and bias across models; Figure S2: Bland–Altman plots for *MD* and *PSD*; Table S3: Clinical and imaging characteristics across quartiles of fellow-eye benefit; Table S4: Fellow-eye contribution scores and their association with Δ*RMSE*; Table S5: Normalized modality weights and their correlations with fellow-eye benefit; Figure S3: Measured and model-predicted mean TS52 maps; Figure S4: Pointwise TS52 error maps; Figure S5: Pointwise TS52 agreement maps; Figure S6: Case-based comparison of fellow-eye interaction using patch-occlusion sensitivity; Table S6: Selected studies predicting 24-2 visual field outcomes from structural, vascular, or biomechanical imaging; Table S7: Exploratory severity-stratified performance of A2 in the test set; Table S8: Comparison of the tabular XGBoost baseline and multimodal A0 on the unchanged test set.
