# Supplementary material for: Multimodal Deep Learning for Humphrey 24-2 Visual Field Prediction: Evaluating the Contribution of Fellow-Eye Information in Glaucoma and Ocular Hypertension
Source: J Clin Med. 2026 Sep 19;15(18):7275. doi: 10.3390/jcm15187275 (PMC13607890; doi:10.3390/jcm15187275)
Supplement: Supplementary file 1 [file jcm-15-07275-s001.zip › jcm-4492710-supplementary.pdf]

Supplementary Materials

1. Supplementary Methods

S1. Quartile Analysis of Fellow-Eye Benefit

The quartile analysis was restricted to eyes from same-visit paired-eye samples for which actual fellow-eye data were available in the test subset. Analyses were conducted separately for A1 and A2. For each eye, fellow-eye benefit was quantified as $\Delta RMSE = RMSE_{OFF} - RMSE_{ON}$ and $\Delta MAE = MAE_{OFF} - MAE_{ON}$.

Positive values indicated lower prediction error when fellow-eye information was enabled. Within each model, eyes were ranked according to *ΔRMSE* and divided into four equal-sized groups. To ensure stable quartile assignment in the presence of tied values, ties were resolved according to their order of occurrence before quartile allocation. Q1 represented the lowest and Q4 the highest fellow-eye benefit.

Clinical and imaging variables included primary-eye MD, primary-eye mean TS52, the absolute intereye MD difference, the Euclidean norm of the intereye differences across the 12 clock-hour RNFL measurements, and the primary-eye OCT image quality score. Mean TS52 was calculated as the average of the 52 analyzed threshold sensitivity values. Quartile characteristics were summarized as median [interquartile range]. The quartile analysis was exploratory and descriptive; no formal between-quartile hypothesis testing was performed.

S2. Fellow-Eye Contribution Scores

Fellow-eye-induced changes in pointwise threshold sensitivity were calculated using predictions obtained under the fellow-eye ON and OFF conditions. For eye *e* and visual field location *k*,

$$\Delta TS_{e,k} = TS_{e,k,ON} - TS_{e,k,OFF}, k = 1, \dots, 52.$$

Three eye-level contribution scores were calculated: the mean absolute pointwise change,

$$mean\ (|\Delta TS|) = \frac{1}{52}\sum_{k=1}^{52}\left|\Delta TS_{e,k}\right|,$$

the 95th percentile of the absolute pointwise changes, and the signed mean pointwise change,

$$mean\ (\Delta TS) = \frac{1}{52}\sum_{k=1}^{52}\Delta TS_{e,k}.$$

Spearman rank correlation analysis was used to evaluate the association between mean */ΔTS/* and *ΔRMSE*. For A2, the model-derived α-gate value was additionally summarized and its association with *ΔRMSE* was assessed using Spearman correlation. These analyses included only paired-eye test samples with actual fellow-eye information.

S3. Modality-Weight Analysis

The quality-aware modality-gating mechanism generated normalized weights for the RNFL thickness map (TM), circular peripapillary RNFL thickness profile (CT), color optic disc photograph (CP), and red-free fundus image (RF). The four weights were obtained using a softmax function and therefore summed to 1 within each eye-level sample.

Modality weights were extracted from the independent test-set predictions and matched to the corresponding eye-level fellow-eye ON/OFF results. Only paired-eye samples with actual fellow-eye data were included. For each model, modality weights were summarized as mean ± standard deviation. Spearman rank correlation coefficients were calculated between each modality weight and *ΔRMSE*.

Because the four modality weights were constrained to sum to 1, their correlations with *ΔRMSE* were not statistically independent. The modality-weight analyses were therefore considered exploratory.

S4. Spatial Mapping and Pointwise Performance Analyses

Pointwise analyses were performed at each of the 52 retained Humphrey 24-2 visual field locations. The two locations corresponding to the physiological blind spot were excluded. All pointwise values were mapped to the right-eye canonical 24-2 layout used during model development.

For each model, the measured and predicted mean threshold sensitivities were calculated separately at every visual field location. Pointwise MAE, RMSE, Lin's concordance correlation coefficient, and coefficient of determination (R^2) were also calculated across test-set eyes at each location. The same display ranges were applied across A0, A1, and A2 for a given metric to facilitate visual comparison.

To characterize the spatial effect of fellow-eye information, $\Delta TS = TS_{ON} - TS_{OFF}$ was calculated at each visual field location in the paired-eye test subset. The mean and standard deviation of *ΔTS* were then calculated across eyes and mapped to the right-eye canonical 24-2 layout. Positive mean *ΔTS* values indicated higher predicted threshold sensitivity when fellow-eye information was enabled.

S5. Case-Based Patch-Occlusion Analysis

Case-based patch-occlusion analyses were performed to explore how the target-eye and fellow-eye thickness maps contributed to TS52 prediction in A1 and A2. Two paired-eye cases were selected for illustrative purposes: one showing relatively preserved intereye structural and functional similarity and one showing marked intereye asymmetry. These cases were not selected to provide population-level inference.

Analyses were performed using the best-performing checkpoint of each model with fellow-eye input enabled. Patch occlusion was applied only to the TM modality. Each TM image was resized to 224×224 pixels, and a 32×32-pixel patch was moved across the image with a stride of 16 pixels. At each position, the patch was replaced by the channel-wise mean value of the corresponding TM image.

Target-eye and fellow-eye TM images were occluded separately. For each patch position, TS52 RMSE was recalculated and compared with the RMSE obtained from the unoccluded image. Patch importance was defined as

$$I_{patch} = RMSE_{occluded} - RMSE_{baseline}.$$

Positive values therefore indicated that occlusion increased prediction error and that the corresponding region contributed to the unoccluded prediction. Importance values were normalized using the 99th percentile of the patch-level scores, restricted to the interval from 0 to 1, resized to the original analysis resolution, and overlaid on the TM image. The patch-occlusion analyses were exploratory and were used only to illustrate model behavior in the selected cases.

S6. Exploratory Severity-Stratified Evaluation

A post hoc severity-stratified analysis was performed using the existing A2 predictions for all 169 test eyes, without retraining, checkpoint reselection, or model modification. Eyes were classified by measured MD using the study definitions: early, MD > −3 dB; mild, −6 dB < MD ≤ −3 dB; moderate, −12 dB < MD ≤ −6 dB; and severe, MD ≤ −12 dB.

Within each stratum, TS52 MAE and RMSE were calculated across all included eyes and all 52 analyzed visual field locations. MAE was also calculated separately for MD and PSD. Percentile 95% confidence intervals were estimated using 2,000 patient-clustered bootstrap resamples with a fixed random seed of 42. Within each severity stratum, patients were sampled with replacement, retaining all eyes belonging to each sampled patient that met the stratum definition. A patient could contribute to more than one stratum when the two eyes differed in severity.

This analysis described absolute prediction performance by severity; it did not estimate the incremental benefit of fellow-eye input within each stratum. No formal between-stratum hypothesis tests were performed.

S7. Conventional Tabular XGBoost Baseline

A post hoc XGBoost baseline was developed using the unchanged patient-level training, validation, and test partitions. The model used 24 target-eye predictors: age, sex, OCT image quality, rim area, disc area, linear and vertical cup-to-disc ratios, cup volume, four quadrant RNFL thickness measurements, and 12 clock-hour RNFL measurements. Neither visual field measurements nor fellow-eye information was included as predictors. Missing predictor values were imputed using means calculated exclusively from the training subset.

Independent regressors were fitted for each of the 52 threshold sensitivities and for MD and PSD. All regressors used a squared-error objective, histogram-based tree construction, a learning rate of 0.05, a minimum child weight of 1, and L2 regularization of 1. Row and column subsampling fractions were both 1.0. Four candidate configurations combined maximum tree depths of 2 or 4 with 150 or 400 boosting trees.

Model selection used validation MAE separately for TS52, MD, and PSD. For TS52, one shared configuration was selected using MAE across all validation eyes and all 52 locations. The selected configuration used a maximum depth of 2 and 150 trees for TS52 and MD, and a maximum depth of 2 and 400 trees for PSD. The selected training-fitted models were retained without refitting on validation data and evaluated on the 169 test eyes from 118 patients.

Performance was compared with A0 using matched test-eye predictions. TS52 MAE was calculated across all eyes and all 52 locations. Pooled TS52 RMSE was calculated as the square root of the mean squared error across these observations, rather than as the average of eye-level RMSE values. MAE was also calculated for MD, PSD, and mean threshold sensitivity, the latter obtained by averaging the 52 measured and predicted sensitivities within each eye.

Percentile 95% confidence intervals for model performance and paired error differences were estimated using 5,000 patient-clustered bootstrap resamples. Identical patient draws were used for both models, retaining all test eyes belonging to each sampled patient. Differences were defined as XGBoost minus A0; negative values indicate lower error for XGBoost. This exploratory comparison did not include equivalence testing.

The analysis used Python 3.10.11, XGBoost 3.1.2, NumPy 1.26.4, and pandas 2.3.1, with a fixed random seed of 42.

2. Supplementary Tables

2.1. Hardware and software environment used for model training and evaluation

The hardware and software environment used for the deep-learning models is summarized in Table S1. The software versions used for the additional XGBoost analysis are reported in Supplementary Methods S7.

Table S1. Hardware and software environment used for model training and evaluation.

Component	Specification
Operating system	Windows 11
Central processing unit	Intel Core i7-13700H
Random-access memory	15.73 GB
Graphics processing unit	NVIDIA GeForce RTX 4050 Laptop GPU, 6 GB
Python	Version 3.12.10
PyTorch	Version 2.5.1+cu121
CUDA	Version 12.1

2.2. Global pairwise differences in MAE and bias across models

Pairwise comparisons of MAE and prediction bias are summarized in Table S2. Differences in MAE were generally small. For MD, no significant differences in MAE were observed, whereas A2 showed significantly higher bias than both A0 and A1. For PSD, A2 had a higher MAE than A1, and A1 showed a more negative bias than A0. For mean threshold sensitivity, both bilateral models showed significantly lower positive bias than A0, whereas the differences in MAE were not significant.

Overall, the effect of bilateral modeling on global outcomes was more evident in prediction bias than in error magnitude.

Table S2. Global pairwise differences in MAE and bias across models for MD, PSD, and mean threshold sensitivity in the test set.

Outcome	Comparison	ΔMAE [95% CI]	p (MAE)	ΔBias [95% CI]	p (Bias)
MD	A1 – A0	-0.08 [-0.28, 0.11]	0.4536	0.10 [-0.08, 0.30]	0.2804
MD	A2 – A0	-0.05 [-0.24, 0.15]	0.6496	0.70 [0.52, 0.89]	<0.0001
MD	A2 – A1	0.03 [-0.13, 0.19]	0.7072	0.60 [0.48, 0.72]	<0.0001
PSD	A1 – A0	-0.06 [-0.14, 0.03]	0.1724	-0.22 [-0.29, -0.15]	<0.0001
PSD	A2 – A0	0.04 [-0.03, 0.12]	0.2784	-0.02 [-0.10, 0.07]	0.6584
PSD	A2 – A1	0.10 [0.03, 0.17]	0.0040	0.20 [0.15, 0.26]	<0.0001
Mean TS	A1 – A0	-0.05 [-0.25, 0.16]	0.6480	-0.59 [-0.85, -0.31]	<0.0001

Mean TS	A2 – A0	-0.12 [-0.33, 0.09]	0.2560	-0.52 [-0.74, -0.26]	<0.0001
Mean TS	A2 – A1	-0.08 [-0.26, 0.10]	0.4072	0.07 [-0.12, 0.27]	0.4048
MD, mean deviation; PSD, pattern standard deviation; mean TS, mean threshold sensitivity; MAE, mean absolute error; CI, confidence interval. Differences were calculated as the first model minus the second model. Negative ΔMAE values indicate lower error in the first model. Bias was calculated as predicted minus measured values. Confidence intervals and p values were estimated using patient-clustered bootstrap resampling.					

2.3. Clinical and imaging characteristics across quartiles of fellow-eye benefit

Table S3 summarizes clinical and imaging characteristics across quartiles of fellow-eye benefit. In A1, the largest positive ΔRMSE values were observed in eyes with less negative MD values, higher mean threshold sensitivity, and relatively limited intereye differences. However, the pattern was not monotonic across all characteristics.

In A2, the quartile with the greatest fellow-eye benefit included eyes with more negative MD, lower mean threshold sensitivity, larger absolute intereye MD differences, and greater RNFL differences. No consistent pattern was observed for image quality.

Table S3. Clinical and imaging characteristics across quartiles of fellow-eye benefit in the paired-eye test subset.

Model and quartile	ΔRMSE, dB	ΔMAE, dB	Primary-eye MD, dB	Primary-eye mean TS, dB	Absolute intereye ΔMD, dB	RNFL difference L2 norm, µm	Primary-eye Image quality
A1 Q1	-0.52 [-0.81, -0.40]	-0.53 [-0.74, -0.39]	-6.44 [-11.04, -4.11]	23.20 [19.46, 24.80]	3.85 [1.60, 8.27]	103.29 [64.34, 136.86]	44.00 [40.00, 46.75]
A1 Q2	-0.07 [-0.13, -0.01]	-0.03 [-0.10, 0.03]	-3.08 [-7.09, -2.22]	27.21 [23.56, 27.67]	1.38 [0.87, 5.13]	68.11 [53.07, 117.11]	46.00 [42.00, 53.00]
A1 Q3	0.17 [0.14, 0.22]	0.20 [0.17, 0.23]	-1.41 [-1.78, -0.93]	29.21 [28.48, 30.46]	0.80 [0.45, 1.39]	67.99 [55.32, 97.76]	48.00 [44.00, 54.00]
A1 Q4	0.70 [0.41, 1.08]	0.60 [0.42, 0.96]	-0.71 [-3.77, 0.21]	30.13 [25.83, 31.49]	1.64 [0.78, 10.24]	71.65 [54.11, 121.70]	44.00 [41.00, 49.00]
A2 Q1	-0.13 [-0.14, -0.09]	-0.09 [-0.11, -0.07]	-1.09 [-2.33, 0.19]	29.23 [27.58, 30.92]	1.12 [0.82, 1.86]	73.97 [54.32, 102.22]	42.00 [40.00, 48.50]
A2 Q2	0.01 [-0.03, 0.02]	0.00 [-0.02, 0.03]	-1.77 [-3.20, -0.39]	29.33 [27.15, 30.79]	1.41 [0.52, 4.47]	62.63 [55.54, 123.61]	47.50 [41.75, 52.50]
A2 Q3	0.14 [0.11, 0.16]	0.09 [0.07, 0.12]	-2.15 [-5.06, -1.30]	27.98 [24.31, 29.52]	1.39 [0.68, 4.58]	67.76 [43.17, 94.94]	46.0 [41.00, 50.00]
A2 Q4	0.27 [0.22, 0.32]	0.22 [0.16, 0.39]	-8.05 [-12.01, -4.46]	22.05 [18.17, 25.70]	4.85 [1.31, 12.81]	109.43 [64.34, 164.21]	46.00 [44.00, 49.00]
Data are presented as median [interquartile range]. *ΔRMSE* and *ΔMAE* were calculated as *OFF − ON*; therefore, positive values indicate lower errors when fellow-eye input was enabled. Q1 represents the lowest and Q4 the highest fellow-eye benefit. RMSE, root mean square error; MAE, mean absolute error; MD, mean deviation; mean TS, mean threshold sensitivity; RNFL, retinal nerve fiber layer.							

2.4. Fellow-eye contribution scores and their association with ΔRMSE

The magnitude of the fellow-eye-induced TS52 change and its association with improvement in RMSE are summarized in Table S4. In A1, mean absolute ΔTS was not significantly associated with ΔRMSE. In A2,

mean absolute ΔTS showed a weak positive correlation with ΔRMSE. The A2 α-gate value was not significantly correlated with prediction improvement.

Table S4. Fellow-eye contribution scores and their association with ΔRMSE in the paired-eye test subset (n = 102 eyes).

Model	Mean $\|\Delta TS\|$, dB	95th percentile of $\|$ΔTS$\|$, dB	Mean ΔTS, dB	*ρ* between mean $\|\Delta TS\|$ and *ΔRMSE* (*p*)	α, mean ± SD	*ρ* between α and *ΔRMSE* (*p*)
A1	0.728 ± 0.656	0.746 ± 0.667	0.382 ± 0.905	0.074 (0.458)	NA	NA
A2	0.503 ± 0.368	1.322 ± 0.829	-0.066 ± 0.064	0.233 (0.018)	0.476 ± 0.063	0.133 (0.182)
TS, threshold sensitivity; RMSE, root mean square error; ΔTS, $TS_{ON} - TS_{OFF}$; ΔRMSE $= RMSE_{OFF} - RMSE_{ON}$; SD, standard deviation; ρ, Spearman correlation coefficient; NA, not applicable. Positive *ΔRMSE* values indicate lower RMSE when fellow-eye information was enabled. For A2, α denotes the model-derived gating coefficient for fellow-eye contribution.						

2.5. Normalized modality weights and their correlations with fellow-eye benefit

Normalized modality weights and their associations with fellow-eye benefit are presented in Table S5. In A1, no modality weight was significantly correlated with ΔRMSE. In A2, the TM and CT weights were positively correlated with ΔRMSE, whereas the CP and RF weights were negatively correlated with ΔRMSE.

Table S5. Normalized modality weights and their correlations with fellow-eye benefit.

Model	TM weight, mean ± SD	*ρ* with *ΔRMSE* (*p*)	CT weight, mean ± SD	*ρ* with ΔRMSE (*p*)	CP weight, mean ± SD	*ρ* with *ΔRMSE* (*p*)	RF weight, mean ± SD	*ρ* with *ΔRMSE* (*p*)
A1	0.2537 ± 0.0002	0.034 (0.733)	0.2506 ± 0.0001	0.094 (0.348)	0.2481 ± 0.0002	-0.057 (0.569)	0.2477 ± 0.0001	-0.025 (0.801)
A2	0.2554 ± 0.0003	0.342 (<0.001)	0.2518 ± 0.0001	0.279 (0.004)	0.2463 ± 0.0002	-0.283 (0.004)	0.2466 ± 0.0002	-0.345 (<0.001)
TM, thickness map; CT, circular peripapillary RNFL thickness profile; CP, color optic disc photograph; RF, red-free fundus image; SD, standard deviation; ΔRMSE, $RMSE_{OFF} - RMSE_{ON}$; ρ, Spearman correlation coefficient. Modality weights were normalized to sum to 1 within each sample.								

2.6. Selected studies predicting 24-2 visual field outcomes from imaging data

Table S6. Selected studies predicting 24-2 visual field outcomes from structural, vascular, or biomechanical imaging

Study	Study sample	Model and inputs	Main reported performance
Guo et al. [5]	112 eyes	Nine-field OCT acquisition with predefined regional structure–function mapping	Mean (r=0.74); mean RMSE = 5.42 dB
Christopher et al. [6]	9765 VF–OCT pairs from 1194 participants and 1909 eyes	RNFL en face images, RNFL thickness maps, and confocal scanning laser ophthalmoscopy images; ResNet-based models	MD MAE = 2.5 dB; PSD MAE = 1.5 dB; AUC = 0.88 for glaucomatous VF damage

Park et al. [7]	2811 training/validation eyes and 290 independent test eyes	Combined macular GCIPL and peripapillary RNFL thickness maps; Inception-V3	Global pointwise RMSE = 4.79 ± 2.56 dB
Lazaridis et al. [8]	Multicenter OCT–VF cohort	Ensemble of deep representation learners using OCT-derived RNFL information	Best model: MAE = 2.3 dB; (R^2=0.78)
Kihara et al. [17]	6437 patients; 5078 image–VF pairs in the test set	OCT and infrared optic disc images; policy-driven multimodal deep learning	Policy-based fusion reduced pointwise MAE relative to individual input branches
Hemelings et al. [18]	1390 OCT–VF pairs from 497 patients	Unsegmented OCT scans; Xception-based regression	MD MAE = 2.89 dB; 52-point MAE = 4.82 dB
Christopher et al. [19]	2999 24-2 VF–OCT pairs from 645 participants and 1222 eyes	Macular retinal layer thickness maps; combined deep learning models	MD MAE = 2.1 dB; PSD MAE = 1.5 dB
Chen et al. [20]	15,026 optic nerve head OCT volumes and 8387 VFs; 996 VF–OCT pairs in the test set	Segmentation-free three-dimensional OCT volume model	Pointwise MAE = 3.11 dB; better performance than two-dimensional OCT maps
Sriwatana et al. [21]	2103 VF–OCT pairs	Artifact-corrected RNFL thickness maps; convolutional and transformer-based models	TS MAE = 3.46 dB; RMSE = 4.44 dB
Mahmoudinezhad et al. [22]	3148 VF–OCTA pairs from 994 participants and 1684 eyes	Optic nerve head OCTA and radial peripapillary capillary images	MD MAE = 1.77 dB; mean pointwise TD MAE = 3.08 dB
Chuangsuwanich et al. [23]	238 participants with glaucoma	Optic nerve head morphology and IOP-induced neural tissue strain; PointNet	F1-score = 0.76 with structural and biomechanical inputs versus 0.71 with structural inputs alone
Current study	591 patients, 892 unique eyes, and 1099 eye-visits	TM, CP, RF, CT, and tabular data; A0 single-eye baseline, A1 bilateral Siamese fusion, and A2 fellow-eye-guided residual refinement	A0: MD MAE/RMSE 2.97/4.40, PSD 1.62/2.30, mean TS 3.26/4.74; A1: 2.90/4.39, 1.56/2.38, 3.21/4.66; A2: 2.93/4.42, 1.66/2.41, 3.13/4.71

The reported outcomes are not directly comparable because the studies differed in study population, visual field target, imaging modality, data partitioning, and performance metric. OCT, optical coherence tomography; VF, visual field; RNFL, retinal nerve fiber layer; GCIPL, ganglion cell–inner plexiform layer; OCTA, optical coherence tomography angiography; IOP, intraocular pressure; TM, thickness map; CP, color optic disc photograph; RF, red-free fundus image; CT, circular peripapillary RNFL thickness profile; MD, mean deviation; PSD, pattern standard deviation; TS, threshold sensitivity; TD, total deviation; MAE, mean absolute error; RMSE, root mean square error; AUC, area under the receiver operating characteristic curve.

2.7. Severity-stratified A2 performance

Supplementary Table S7 summarizes A2 prediction performance according to measured visual field severity. Absolute errors were higher in the moderate and severe groups than in the early and mild

groups. The severe group comprised 22 eyes from 20 patients. These exploratory results indicate that the overall test-set estimates do not adequately characterize performance in advanced disease.

Supplementary Table S7. Exploratory severity-stratified performance of A2 in the test set.

Severity	Patients / eyes, n	TS52 MAE, dB	TS52 RMSE, dB	MD MAE, dB	PSD MAE, dB
Early	70 / 91	2.63 [2.14–3.31]	4.24 [2.86–5.79]	1.97 [1.38–2.71]	1.04 [0.83–1.28]
Mild	36 / 38	2.79 [2.41–3.20]	4.31 [3.43–5.25]	2.43 [1.85–3.07]	1.53 [1.01–2.12]
Moderate	15 / 18	5.45 [4.67–6.40]	7.77 [6.44–9.17]	4.08 [2.94–5.21]	3.01 [2.10–4.20]
Severe	20 / 22	10.13 [8.96–11.39]	12.47 [11.19–13.70]	6.81 [5.01–8.66]	3.37 [2.46–4.29]

Values are estimates [95% confidence intervals]. Severity was defined using measured MD: early, MD > −3 dB; mild, −6 dB < MD ≤ −3 dB; moderate, −12 dB < MD ≤ −6 dB; and severe, MD ≤ −12 dB. Confidence intervals were estimated using 2,000 patient-clustered bootstrap resamples within each stratum. Patients could contribute eyes to different strata; therefore, patient counts across rows are not additive. TS52 errors were calculated across all eyes and all 52 analyzed locations within each stratum, rather than from the difference between measured and predicted mean threshold sensitivity. Both paired and unpaired test eyes were included. MD, mean deviation; PSD, pattern standard deviation; TS52, 52-point threshold sensitivity; MAE, mean absolute error; RMSE, root mean square error.

2.8. Comparison with a conventional tabular baseline

Supplementary Table S8 compares the tabular XGBoost baseline with the multimodal single-eye model A0 on the identical test subset. XGBoost yielded numerically lower errors for all evaluated metrics, but all paired difference confidence intervals included zero. The comparison provides a conventional reference without establishing superiority or equivalence.

Supplementary Table S8. Comparison of the tabular XGBoost baseline and multimodal A0 on the unchanged test set.

Outcome	Metric	A0 [95% CI]	XGBoost [95% CI]	Difference [95% CI]
TS52	MAE	3.99 [3.41–4.60]	3.92 [3.38–4.51]	−0.07 [−0.29–0.17]
TS52	Pooled RMSE	6.38 [5.47–7.25]	6.16 [5.27–7.02]	−0.23 [−0.53–0.10]
MD	MAE	2.97 [2.46–3.51]	2.88 [2.37–3.43]	−0.09 [−0.38–0.23]
PSD	MAE	1.62 [1.34–1.92]	1.55 [1.26–1.88]	−0.07 [−0.24–0.10]
Mean TS	MAE	3.26 [2.72–3.83]	3.00 [2.47–3.56]	−0.25 [−0.55–0.08]

All values are in dB. Both models were evaluated on the same 169 eyes from 118 patients. Differences were calculated as XGBoost minus A0; negative values indicate lower error for XGBoost. Percentile 95% confidence intervals were estimated using 5,000 paired patient-clustered bootstrap resamples. TS52 MAE and pooled RMSE summarize errors across all eyes and all 52 locations; mean TS MAE summarizes the error in the eye-level average sensitivity. XGBoost used target-eye tabular predictors, whereas A0 additionally used images. This comparison therefore does not isolate architectural effects. TS52, 52-point threshold sensitivity; MD, mean deviation; PSD, pattern standard deviation; mean TS, mean threshold sensitivity; MAE, mean absolute error; RMSE, root mean square error; CI, confidence interval.

3. Supplementary Figures

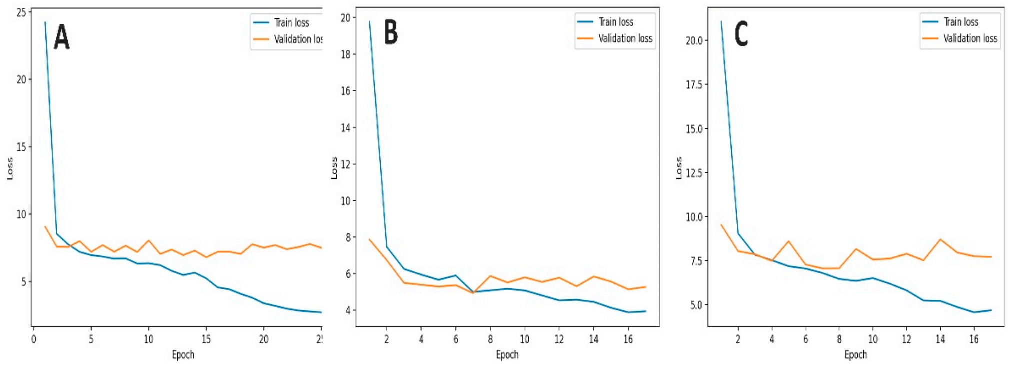

Figure S1. Epoch-level training and validation loss curves for the A0, A1, and A2 models. Panels (A–C) show the A0, A1, and A2 models, respectively. Each panel presents the total training and validation loss across epochs. For A0 and A1, the total optimization loss comprised the weighted combination of Huber loss for TS52 and mean squared error losses for MD and PSD. For A2, the loss additionally included regularization terms for the α-gate magnitude and the α-scaled bounded residual correction. Training was terminated after 10 consecutive epochs without improvement in validation loss, and the checkpoint associated with the lowest validation loss was retained for final test-set evaluation. The best-performing checkpoints were obtained at epoch 15 for A0 and epoch 7 for both A1 and A2.

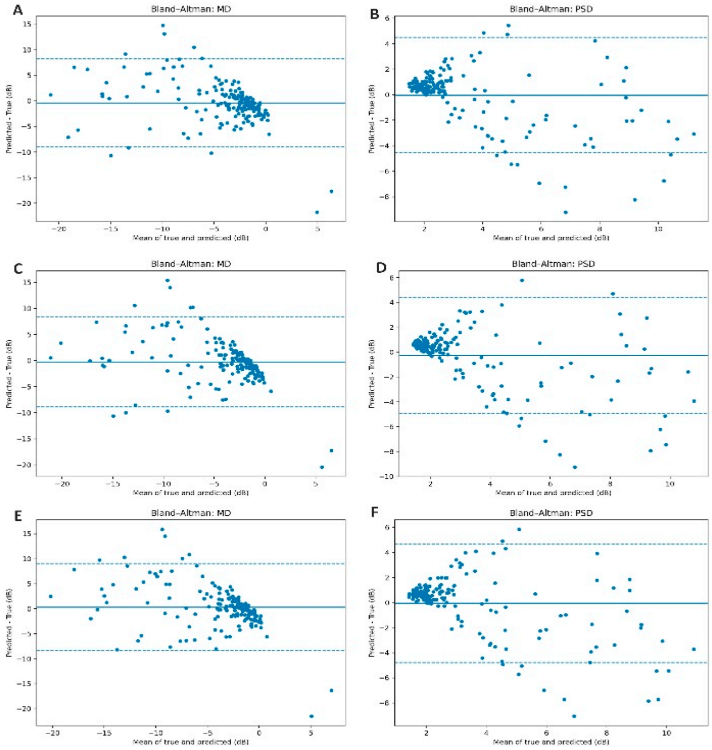

Figure S2. Bland–Altman plots for MD and PSD in the A0, A1, and A2 models on the test set. Rows correspond to A0, A1, and A2, and columns correspond to MD and PSD. The horizontal axis represents the mean of the measured and predicted values, and the vertical axis represents the prediction

difference, calculated as predicted minus measured. The solid line indicates the mean bias, and the dashed lines indicate the 95% limits of agreement.

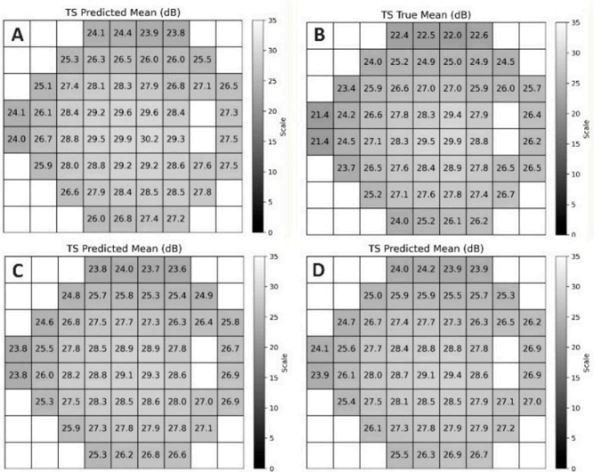

Figure S3. Measured and model-predicted mean TS52 maps. The maps show (A) measured mean TS52 and predicted mean TS52 for (B) A0, (C) A1, and (D) A2 in the right-eye canonical 24-2 layout. All panels are displayed using the same scale. The three models preserved the overall central and paracentral pattern but showed compression of the predicted sensitivity range at locations with lower measured sensitivity.

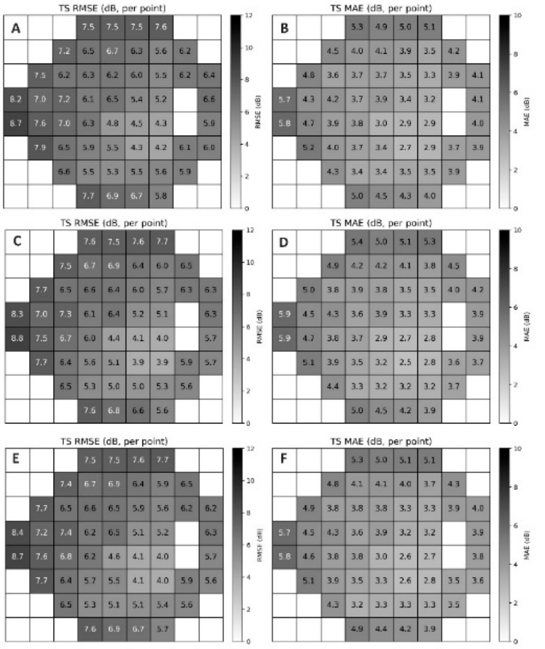

Figure S4. Pointwise TS52 error maps for A0, A1, and A2 on the test set. Panels (A,B), (C,D), and (E,F) show RMSE and MAE maps for A0, A1, and A2, respectively. Values are presented for each TS52 location in the right-eye canonical 24-2 layout. Errors were generally higher at peripheral and lower-sensitivity locations.

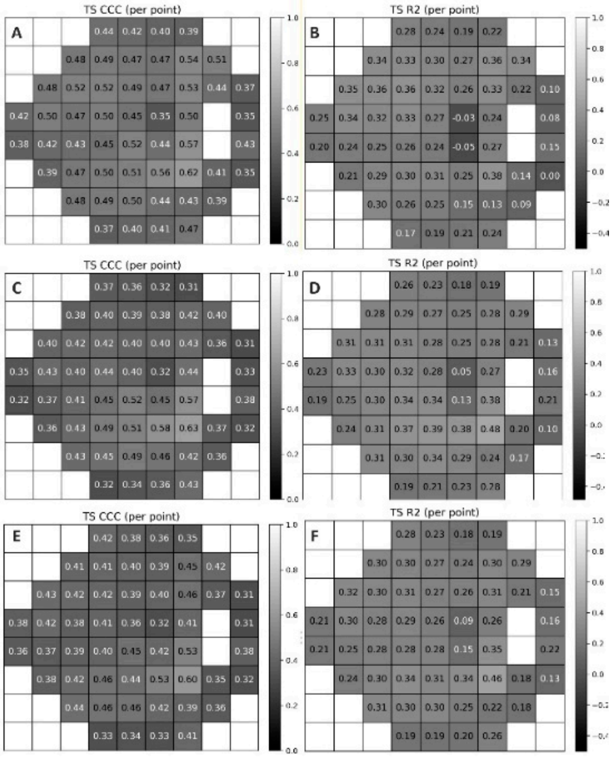

Figure S5. Pointwise TS52 agreement maps for A0, A1, and A2 on the test set. Panels (A,B), (C,D), and (E,F) show CCC and R^2 maps for A0, A1, and A2, respectively. Values are presented for each TS52 location in the right-eye canonical 24-2 layout. Agreement was generally higher at central and paracentral locations and lower at selected peripheral locations.

Case-Based Patch-Occlusion Results

Figure S6 presents two illustrative paired-eye cases. In Case 1, enabling fellow-eye input increased A1 RMSE in the more severely affected right eye from 2.37 to 2.75 dB, whereas it reduced A2 RMSE from 3.01 to 2.80 dB. In Case 2, A1 reduced prediction error in the more severely affected right eye but increased RMSE in the better-preserved left eye from 4.83 to 5.44 dB. In contrast, A2 reduced RMSE in both eyes, including a reduction from 4.93 to 4.81 dB in the left eye. Patch-occlusion maps showed a more diffuse distribution of importance in A1 and a more compact distribution in A2, with greater emphasis on target-eye inputs in the selected examples.

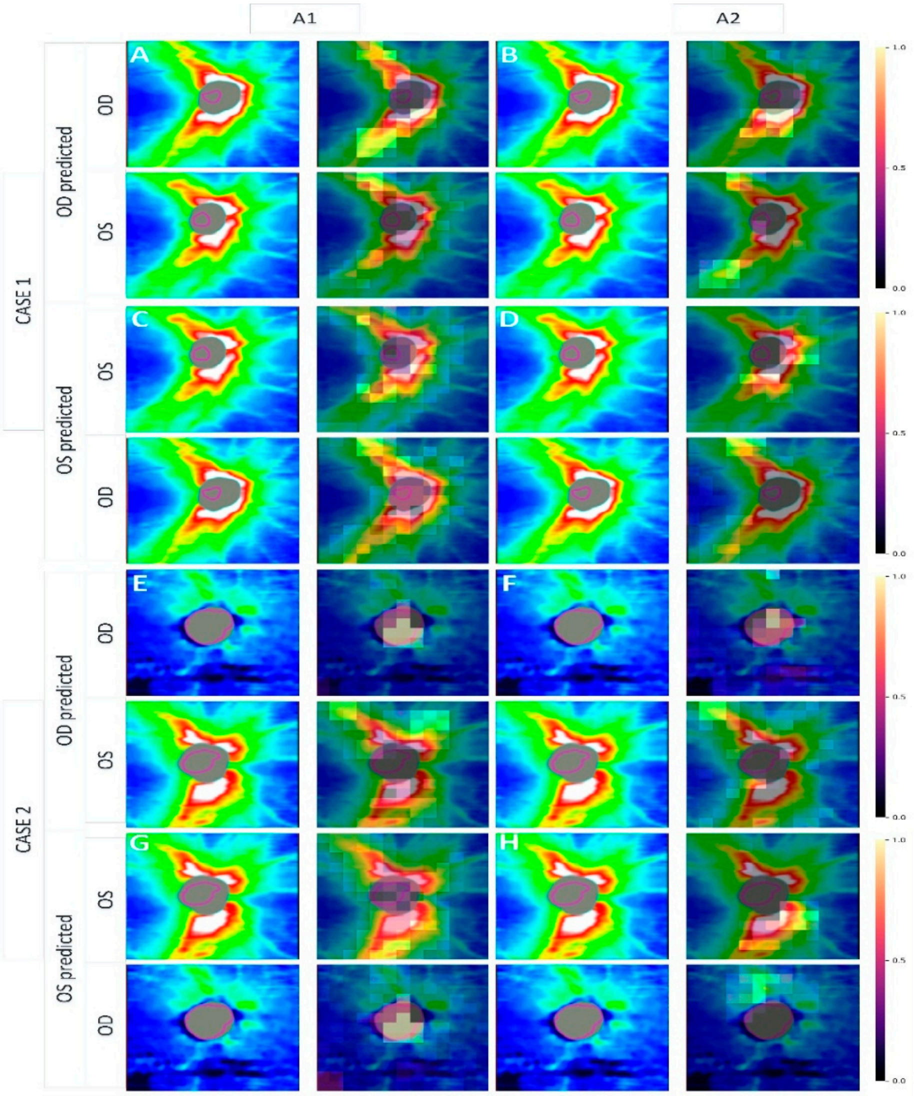

Figure S6. Case-based comparison of fellow-eye interaction in A1 and A2 using patch-occlusion sensitivity on thickness-map images. The upper panel shows a relatively symmetric paired-eye case, and the lower panel shows a case with marked intereye asymmetry. For each case, the left block presents A1 and the right block presents A2. Within each model block, the first column shows the original thickness map and the second column shows the corresponding patch-occlusion importance overlay. The upper two rows show predictions for the right eye and the lower two rows show predictions for the left eye. RMSE values are reported for the fellow-eye OFF and ON conditions. The examples were selected for illustrative purposes and do not represent population-level inference.
